# Supplementary figures and images for: Mammary gland multi-omics data reveals new genetic insights into milk production traits in dairy cattle
Source: PLoS Genet. 2025 Apr 17;21(4):e1011675. doi: 10.1371/journal.pgen.1011675 (PMC12054919; doi:10.1371/journal.pgen.1011675)

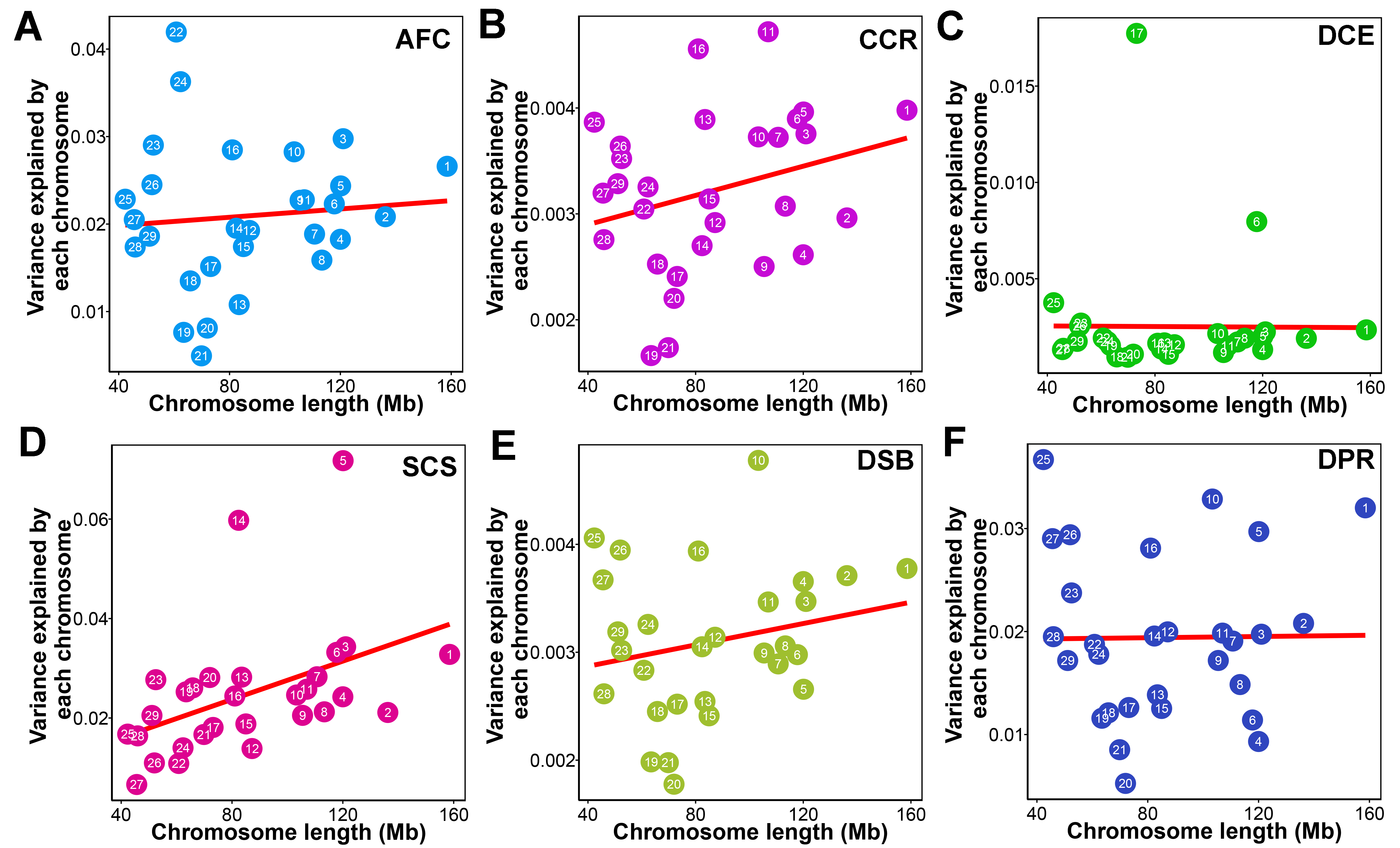

Supplement: S1 Fig — The proportion of variance explained by each chromosome against chromosome length is shown for (A) age at first calving (AFC), (B) cow conception rate (CCR), (C) daughter calving ease (DCE), (D) somatic cell score (SCS), (E) daughter still birth (DSB), and (F) daughter pregnancy rate (DPR) by joint analysis. The numbers in the circles and squares are the chromosome numbers. The regression adjusted R2 (P-value) were -0.029 (0.65) for AFC, 0.044 (0.14) for CCR, -0.037 (0.97) for DCE, 0.074 (0.083) for SCS, 0.013 (0.25) for DSB, and -0.037 (0.95) for DPR, respectively. The variance explanation of each chromosome for heifer conception rate (HCR) was not shown here, due to its Log-likelihood analysis was not converged. (TIF) [file pgen.1011675.s001.tif]

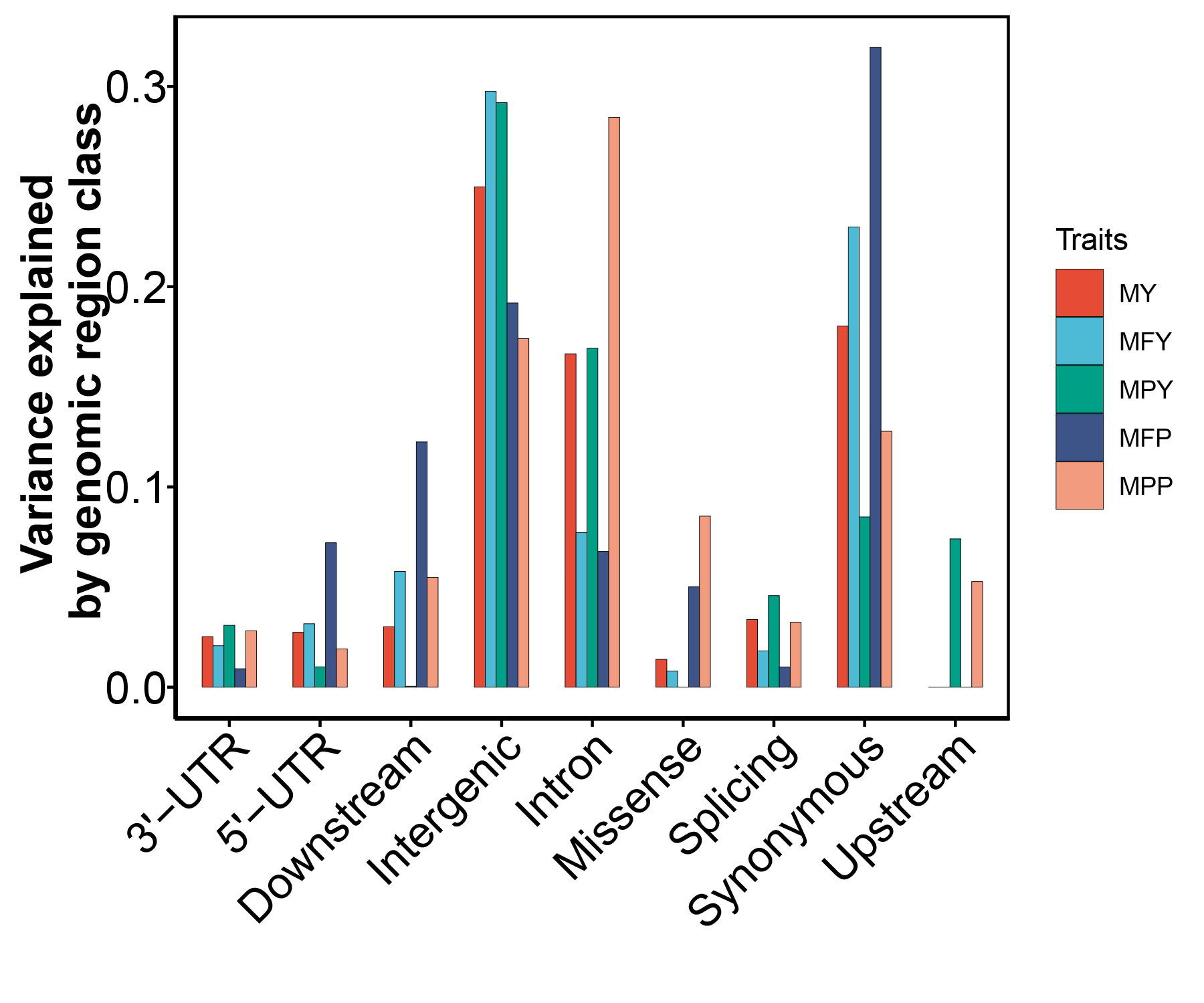

Supplement: S2 Fig — (TIF) [file pgen.1011675.s002.tif]

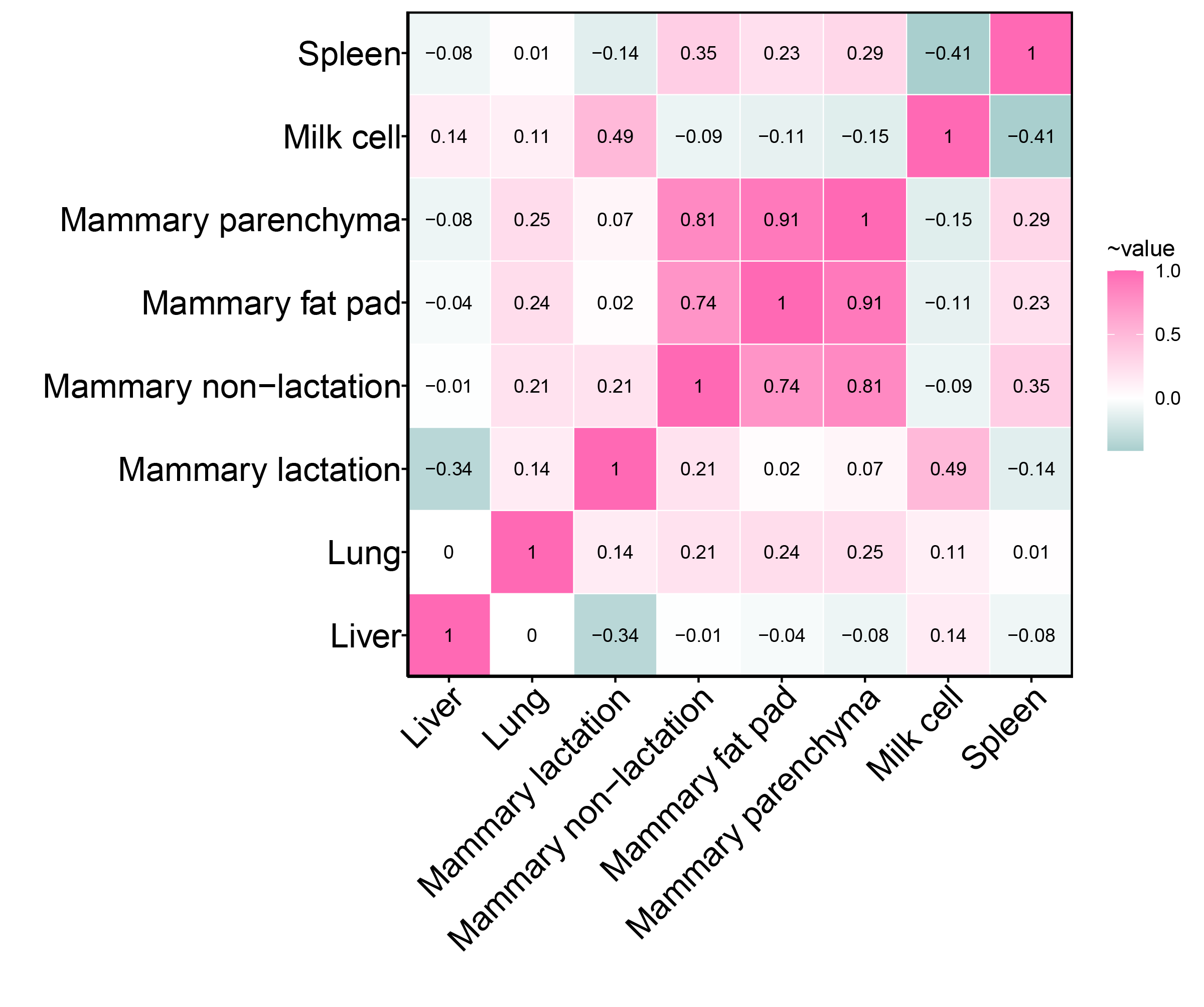

Supplement: S3 Fig — (TIF) [file pgen.1011675.s003.tif]

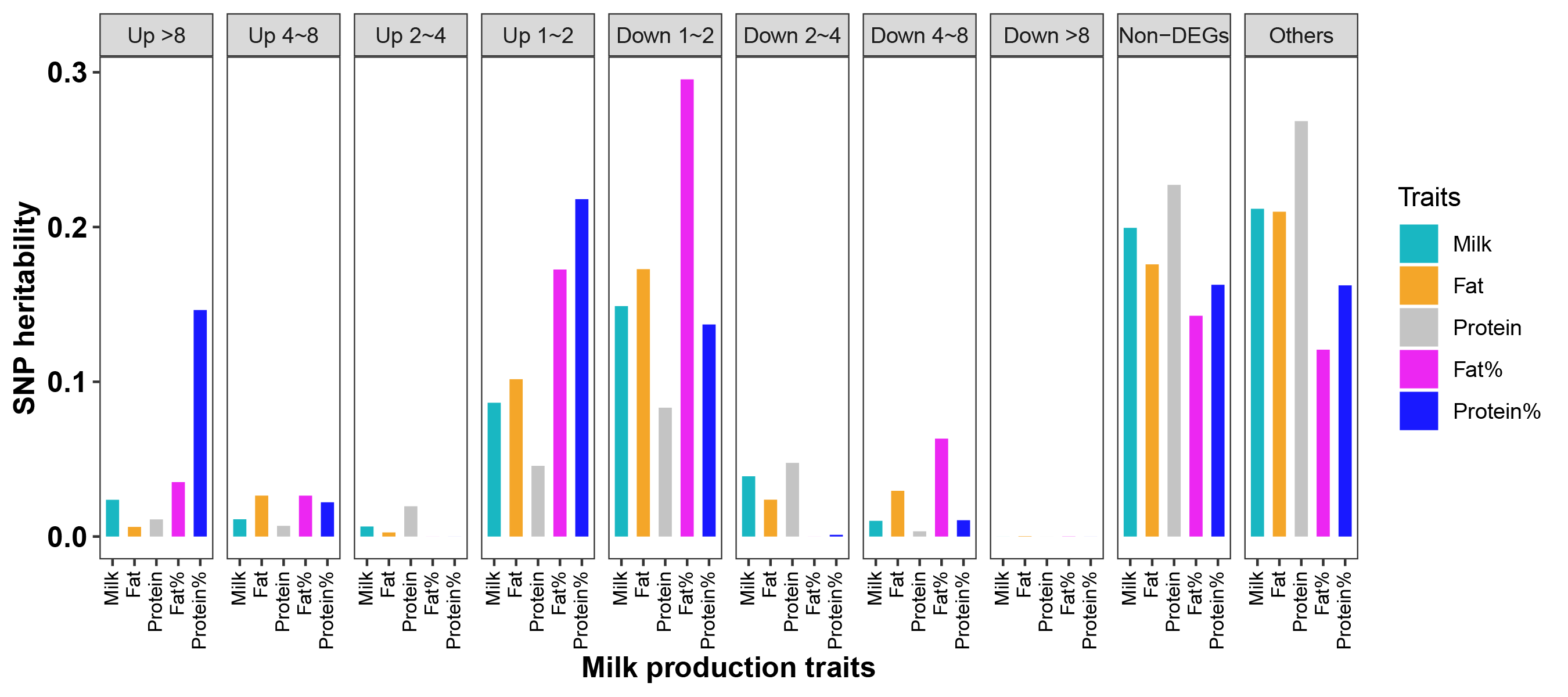

Supplement: S4 Fig — (TIF) [file pgen.1011675.s004.tif]

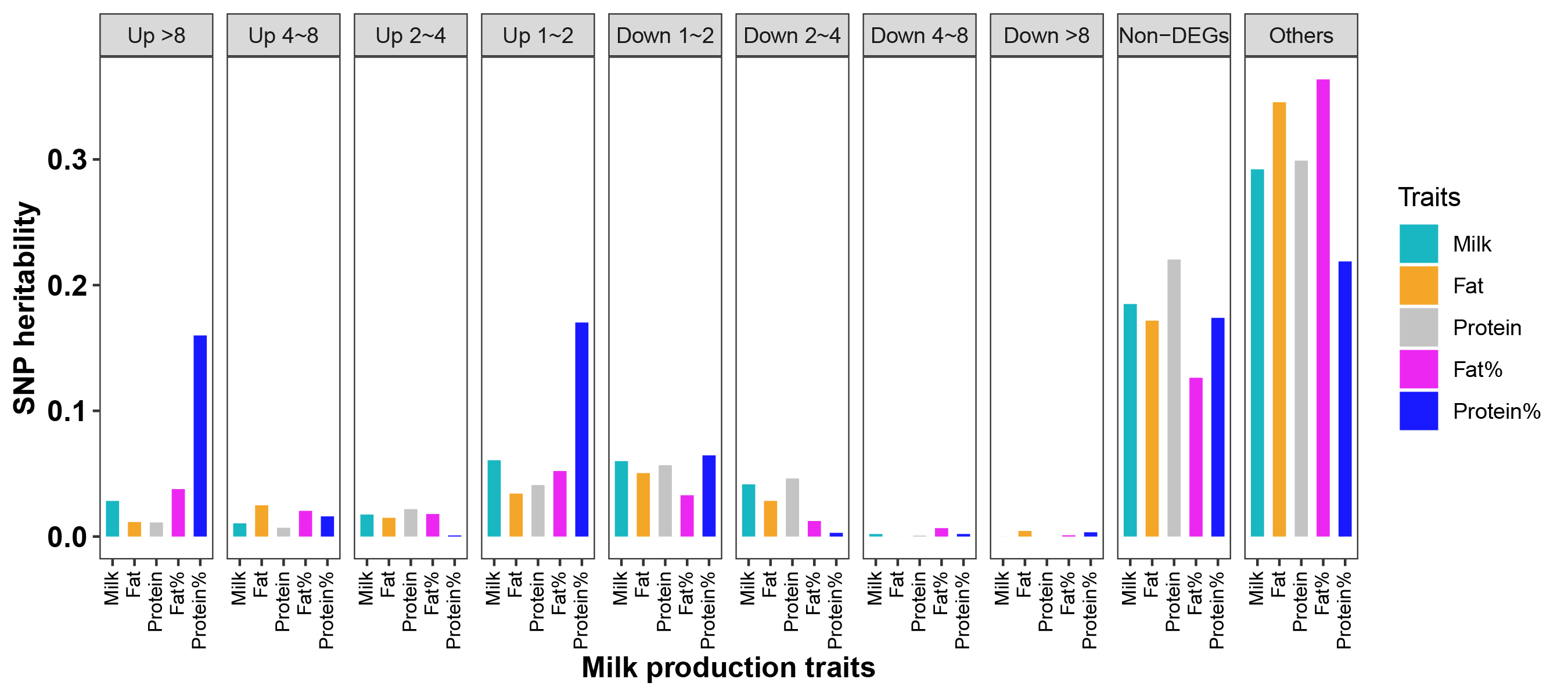

Supplement: S5 Fig — (TIF) [file pgen.1011675.s005.tif]

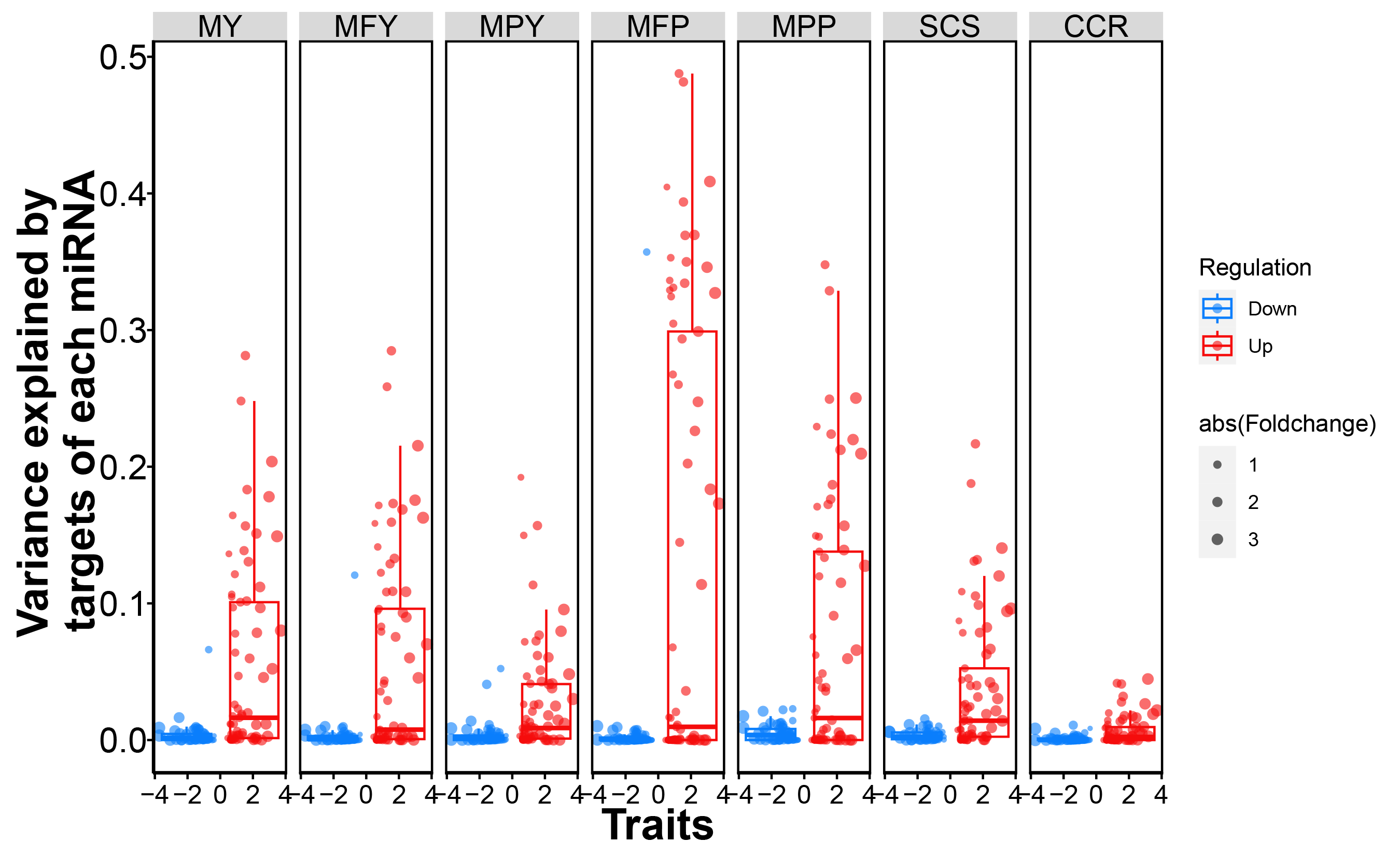

Supplement: S6 Fig — The red and blue colors of point represent the down and up-regulated miRNAs. The size of point represents the absolute value of fold change. (TIF) [file pgen.1011675.s006.tif]

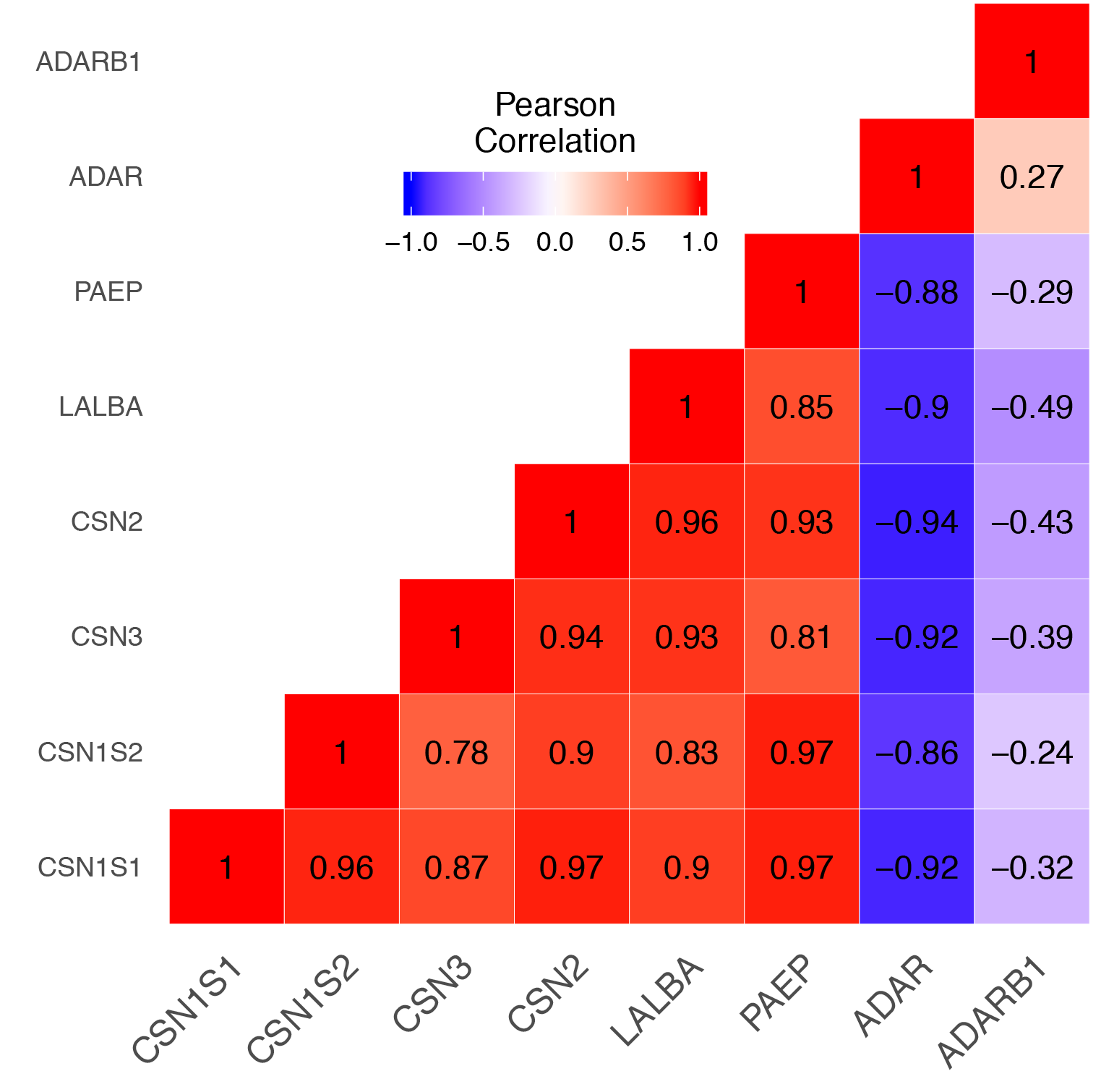

Supplement: S7 Fig — (TIF) [file pgen.1011675.s007.tif]

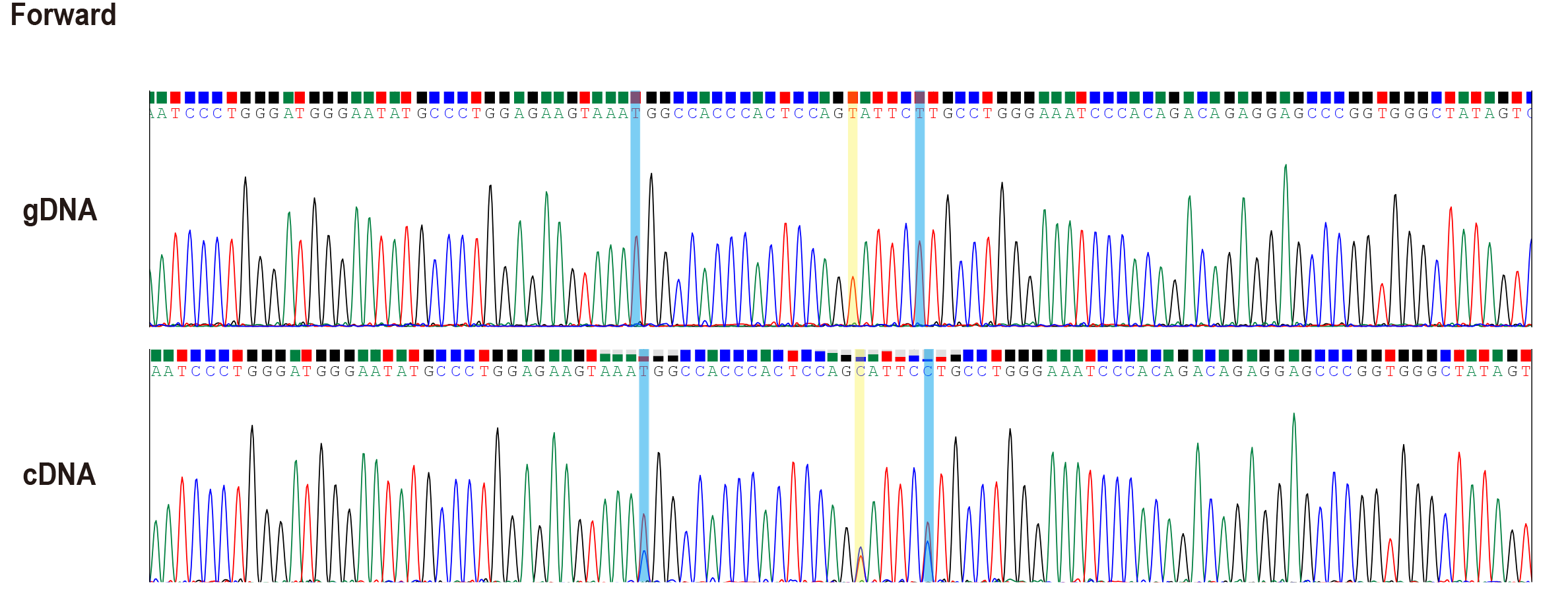

Supplement: S8 Fig — The validated editing sites were marked with yellow background. The novel editing sites were marked with blue background. (TIF) [file pgen.1011675.s008.tif]

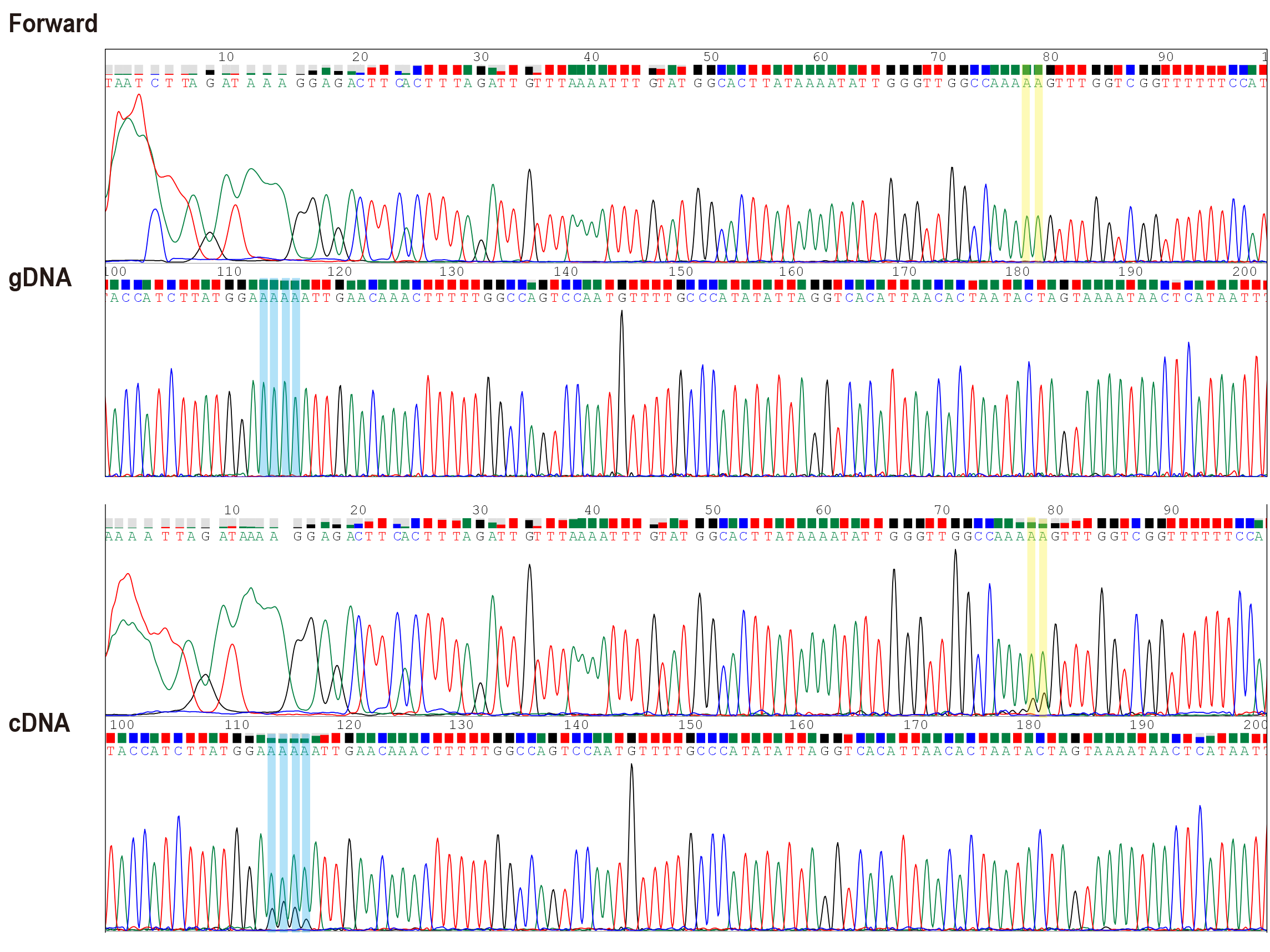

Supplement: S9 Fig — The validated editing sites were marked with yellow background. The novel editing sites were marked with blue background. (TIF) [file pgen.1011675.s009.tif]

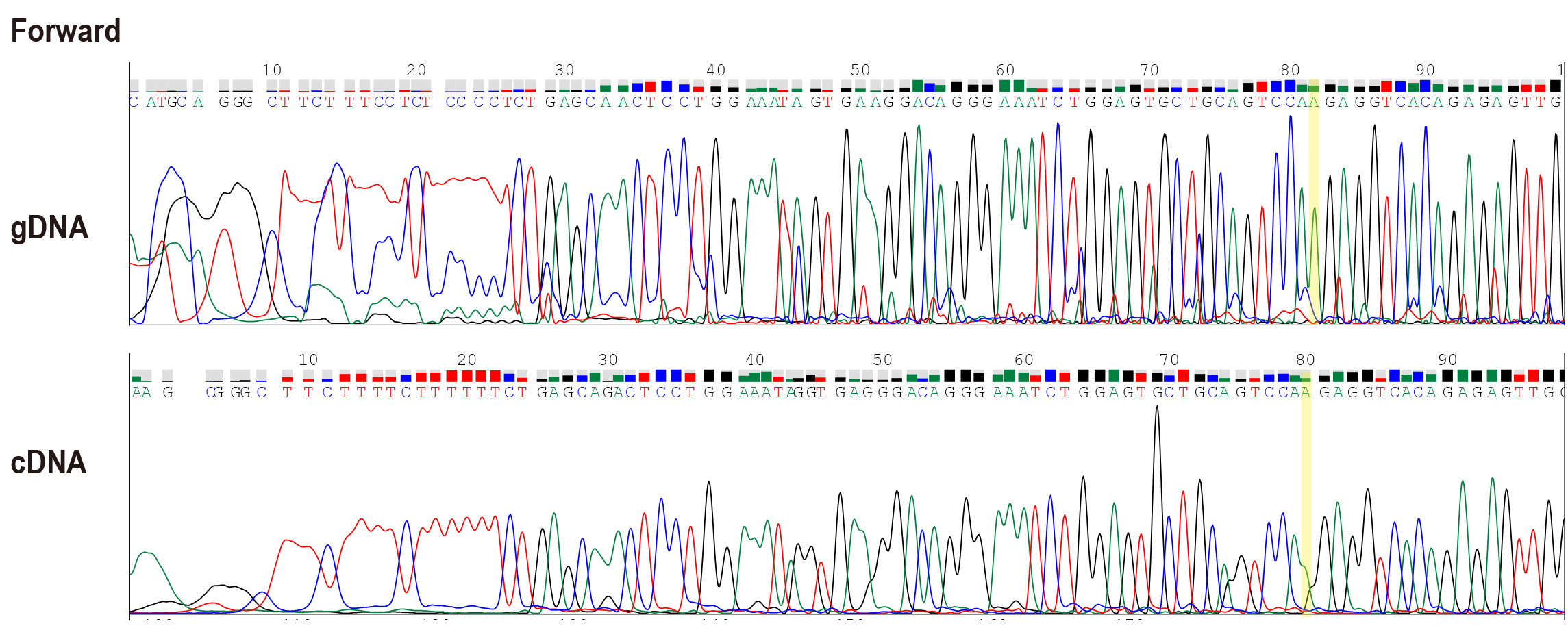

Supplement: S10 Fig — The validated editing sites were marked with yellow background. (TIF) [file pgen.1011675.s010.tif]

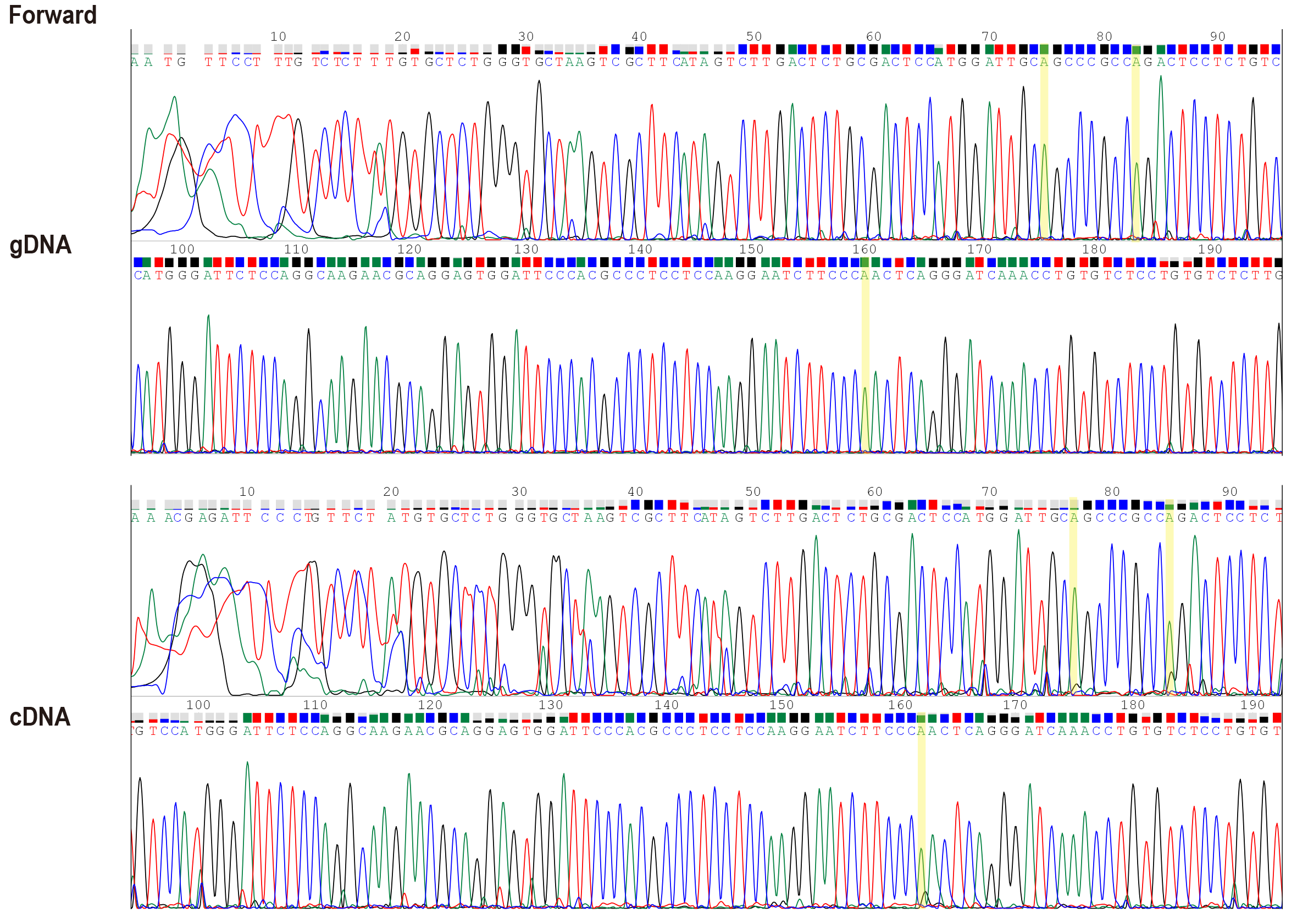

Supplement: S11 Fig — The validated editing sites were marked with yellow background. (TIF) [file pgen.1011675.s011.tif]

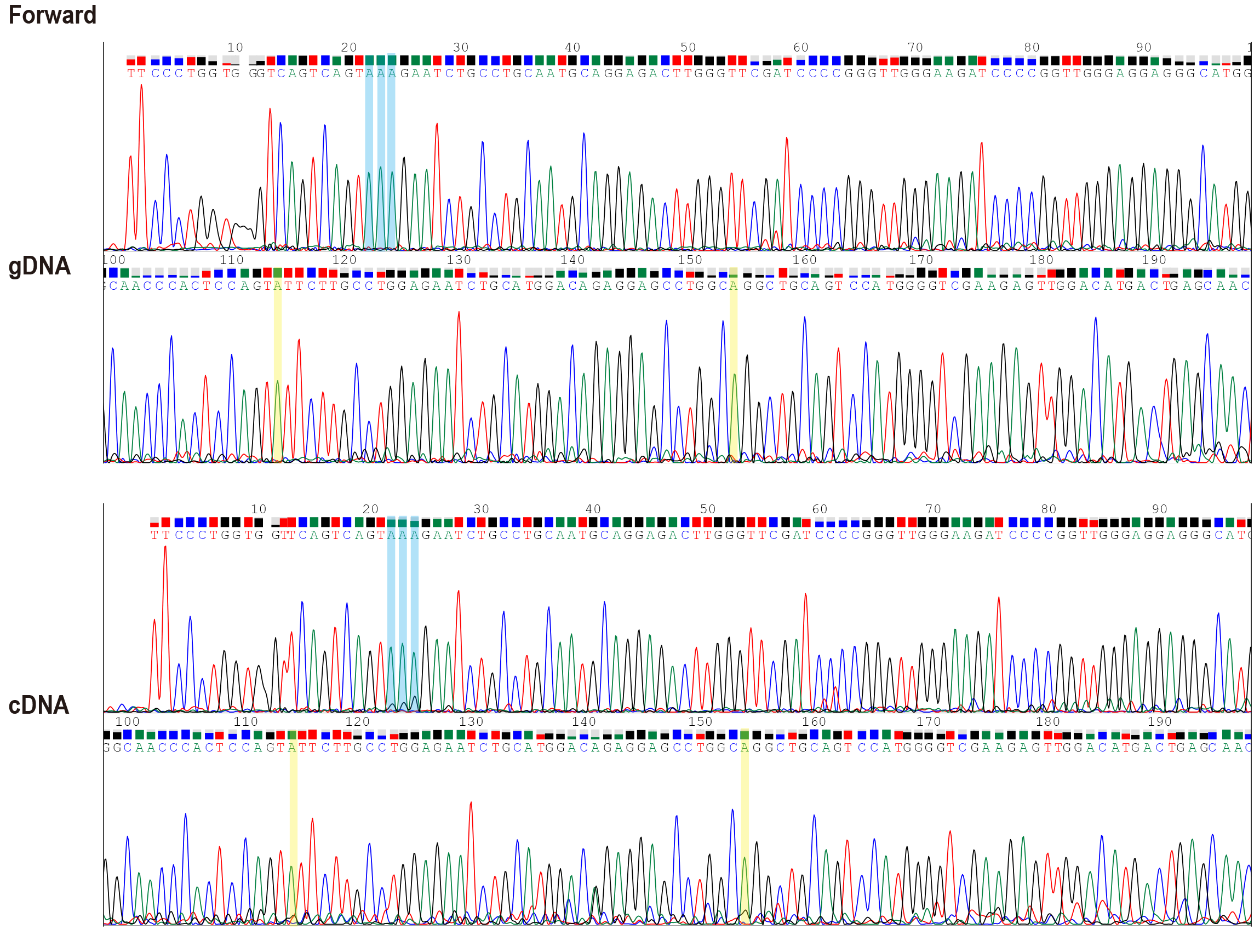

Supplement: S12 Fig — The validated editing sites were marked with yellow background. The novel editing sites were marked with blue background. (TIF) [file pgen.1011675.s012.tif]

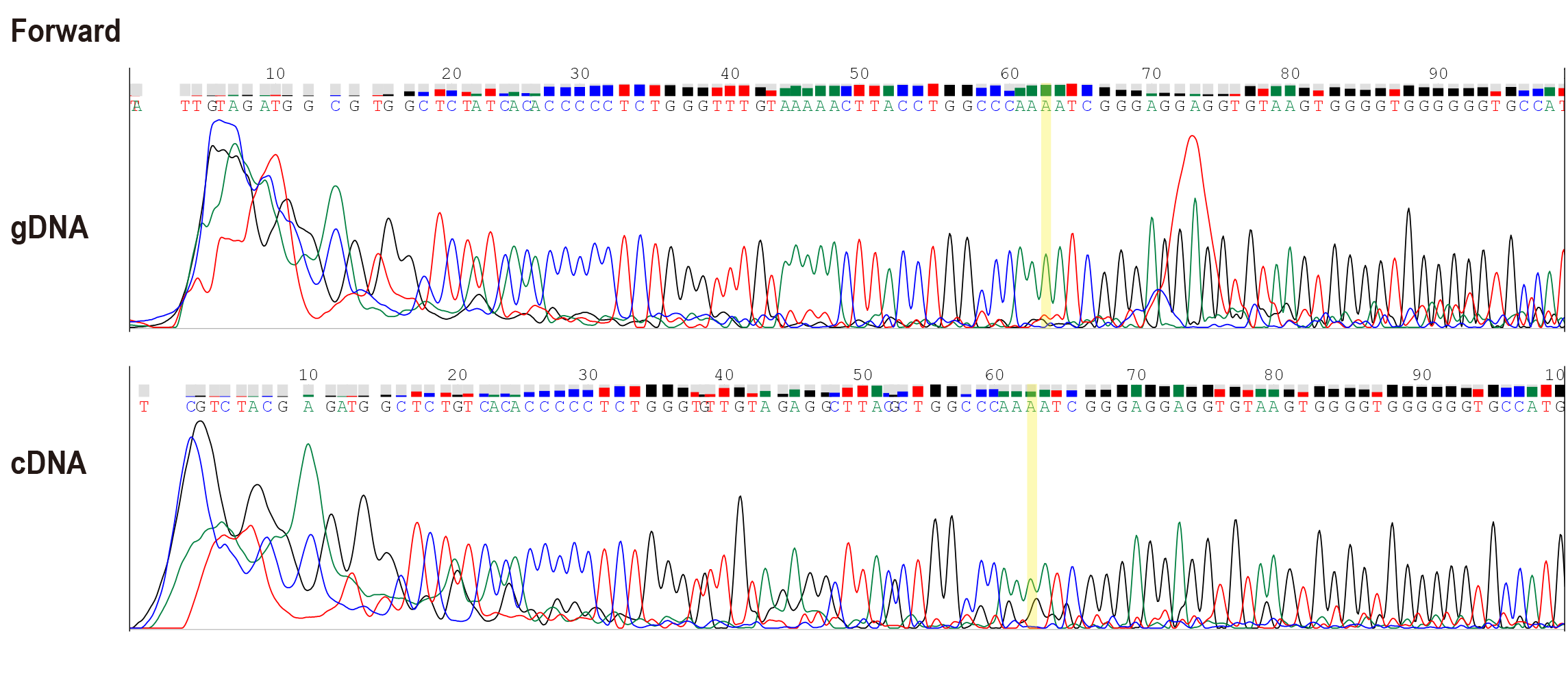

Supplement: S13 Fig — The validated editing sites were marked with yellow background. (TIF) [file pgen.1011675.s013.tif]

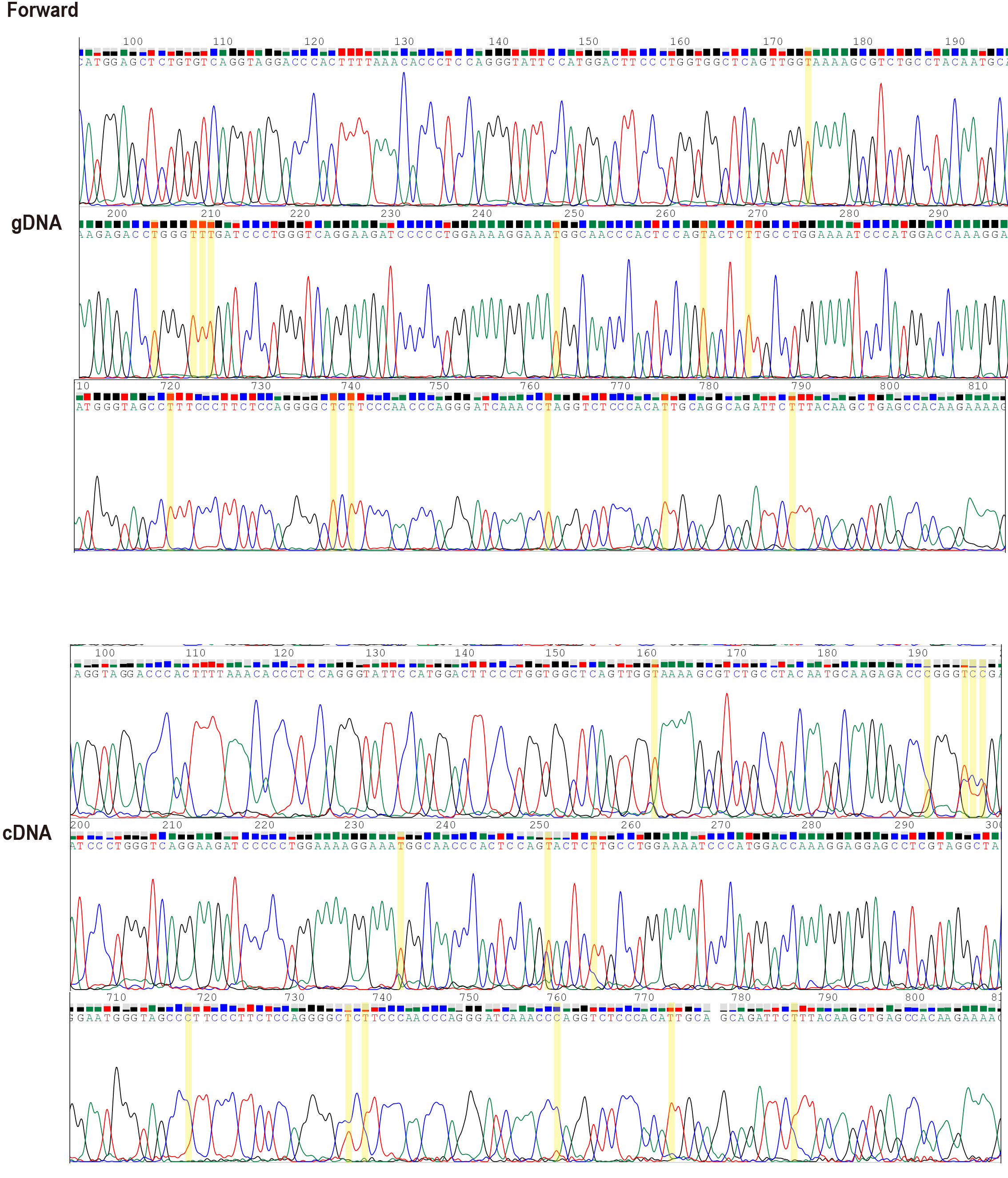

Supplement: S14 Fig — The validated editing sites were marked with yellow background. (TIF) [file pgen.1011675.s014.tif]

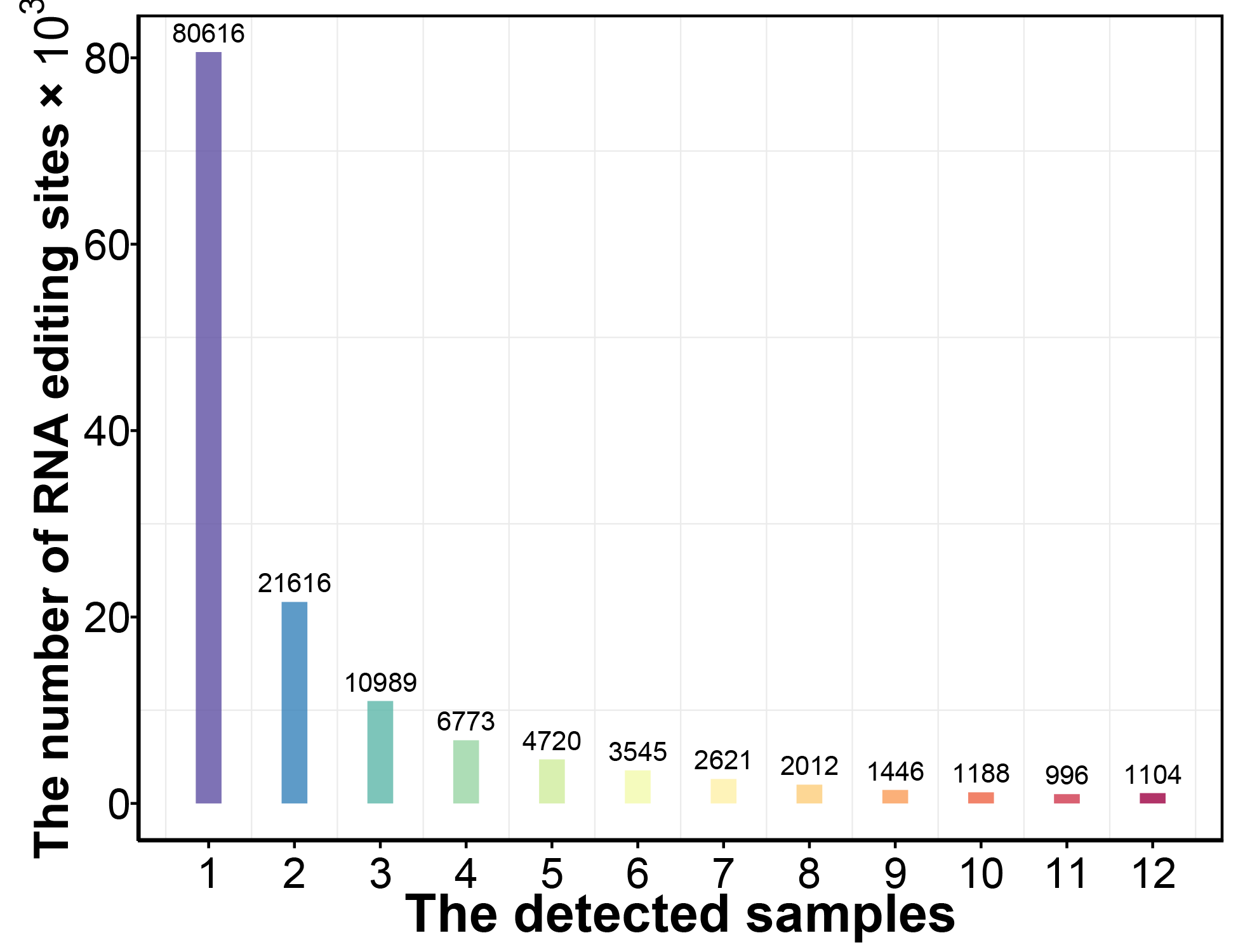

Supplement: S15 Fig — (TIF) [file pgen.1011675.s015.tif]

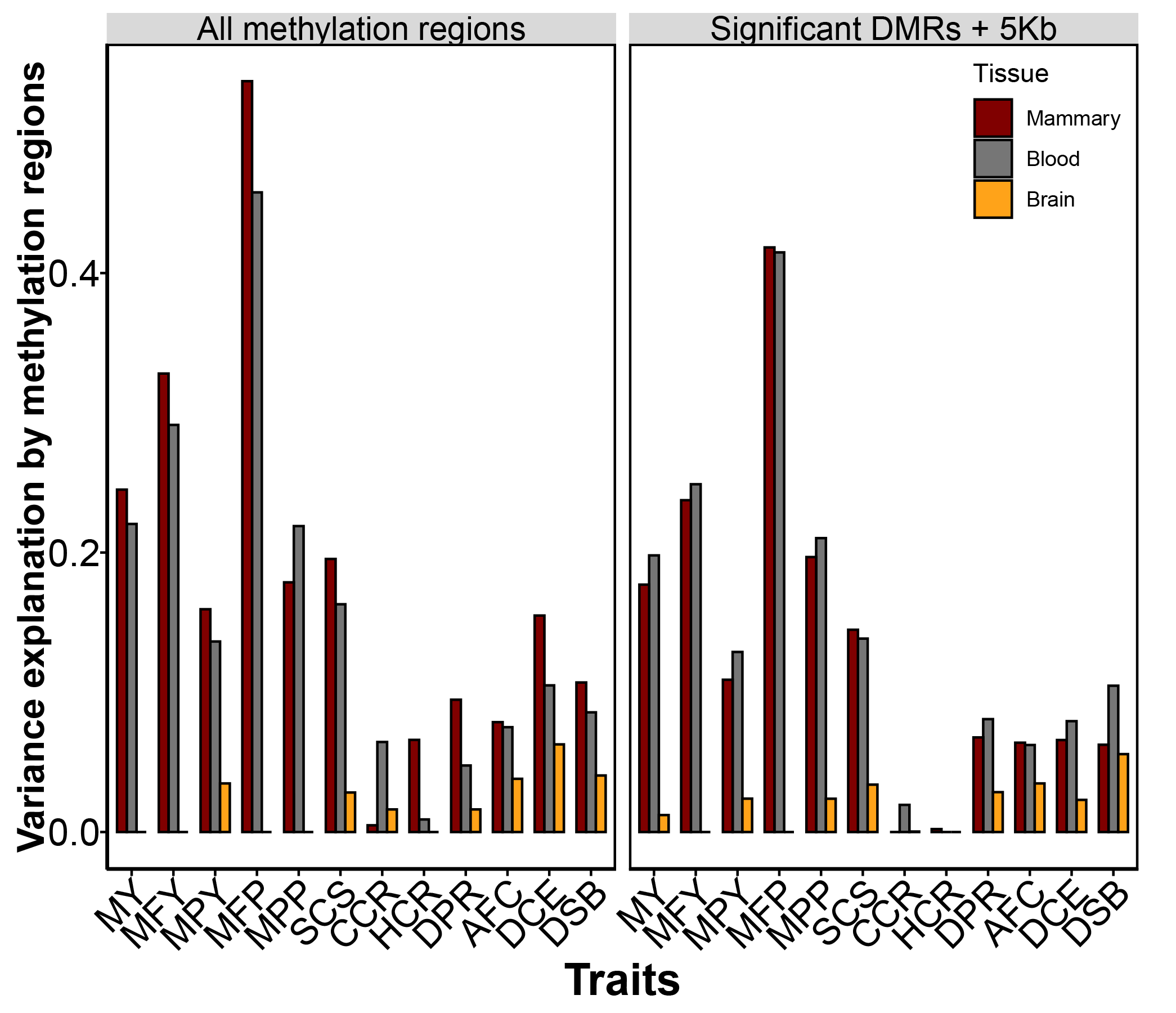

Supplement: S16 Fig — (TIF) [file pgen.1011675.s016.tif]

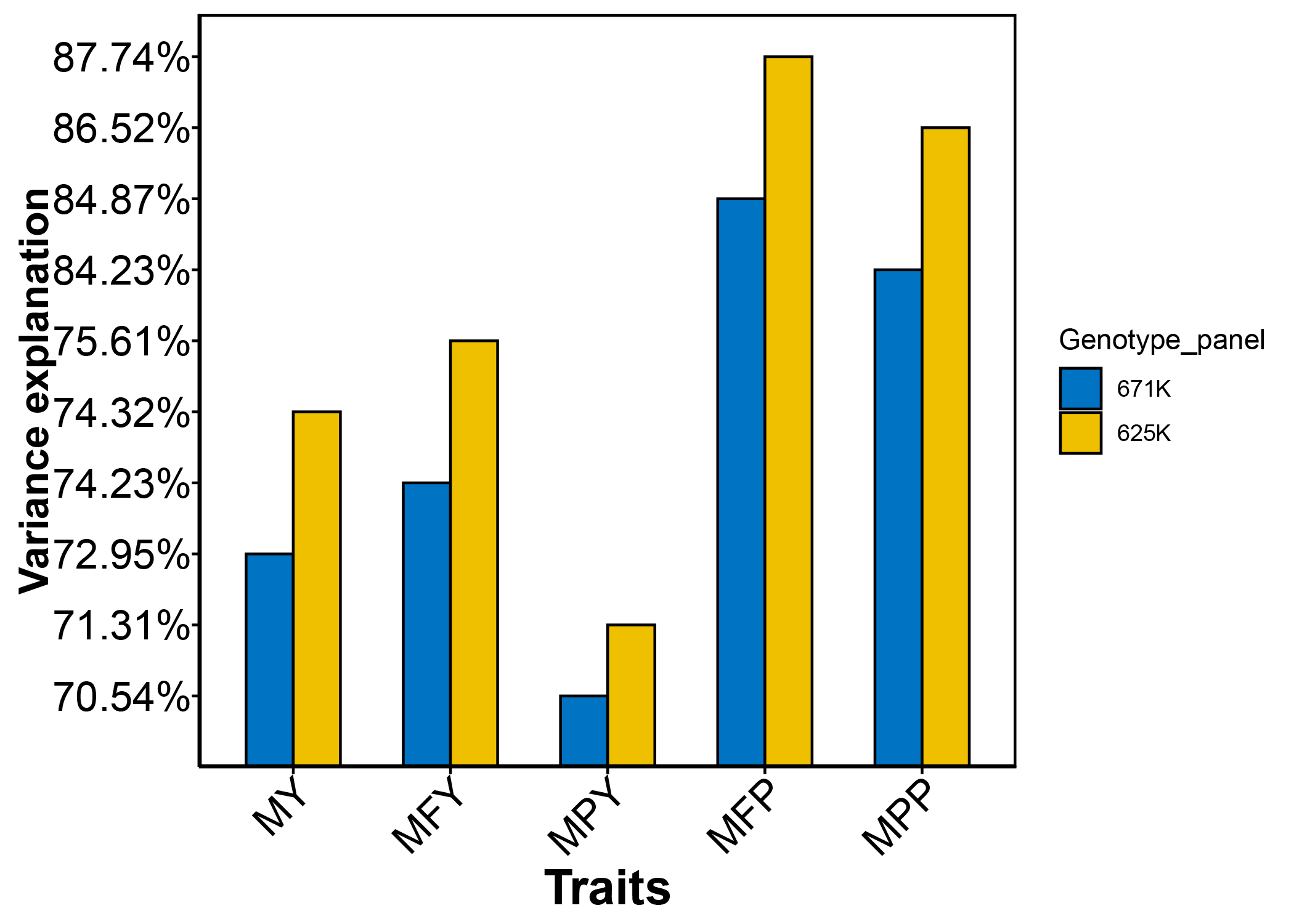

Supplement: S17 Fig — (TIF) [file pgen.1011675.s017.tif]
