# Supplementary material for: Mammary gland multi-omics data reveals new genetic insights into milk production traits in dairy cattle
Source: PLoS Genet. 2025 Apr 17;21(4):e1011675. doi: 10.1371/journal.pgen.1011675 (PMC12054919; doi:10.1371/journal.pgen.1011675)
Supplement: Attachment — Submitted filename: Review_PLOSGenetics_091324.docx [file pgen.1011675.s028.docx]

I have reviewed the manuscript titled **‘Mammary gland multi-omics data reveals new genetic insights into milk production traits in dairy cattle’** by Cai et al. The manuscript is interesting. The introduction is good, easily introduces the reader to the topic, and identifies the gaps in knowledge to fill. The results and discussion sections are long and sometimes hard to follow. I have major comments for the authors.

**Major comments**

The results section is long, and sometimes, it is hard to follow the amount of results the authors showed. I propose the authors remove the section about functional verification of miRNAs that shows up in the manuscript disconnected from the other results (even if I understand the relevance of this verification), but those results contribute little to the manuscript. Also, in the materials and methods section, this functional verification is at the end and disconnected from the previous paragraph. The authors have many relevant results to emphasize, and I think that extra results only increase the complexity of the manuscript.

The authors used deregressed PTAs as the milk production phenotypes. I am concerned about the potential impact on the results obtained. The authors showed a large genetic variance explained for those phenotypes larger than we expected using DTD.

**Minor comments**

L49: Please remove ‘(eQTLs and sQTLs)’.

L64: Please change ‘dairy research’ to ‘animal genetics research’ to give a broader idea of the relevance of the high-throughput technologies.

L72: Please remove the extra blank space after ‘[11],’.

L93-99: Please remove lines 93 to 99. This part is extra information of only one of the functional classes that does not contribute much to the paragraph per se. Or, if authors want to keep it, it could be good to have more examples for different functional classes and not only for RNA editing.

L119-125: Please rewrite. I suggested the following: ‘This study of the mammary gland aims to 1) identify and define the sequencing segments …; 2) determine which functional indicators …; and 3) assess the increase in accuracy ….’.

L126-128: Please remove these lines. They should be placed in the discussion or conclusion sections.

L131: Please change the subtitle ‘Genome partitioning of genetic variation by autosomes and genomic regions’ to ‘Genome partitioning of genetic variation’.

L146-149/ L290-291: Please do not include any references in the results section. These lines should be in the discussion section.

L168: Please remove the extra punctuation you have in ‘(Figure. S3)’.

L170-172: These line could be shortened by combine in one like this ‘As expected, the lactating mammary gland-specific genes explained more genetic variance of milk production (MFP and MPP) than the other four mammary tissues (Table 1). Otherwise, in non-mammary tissues, …’.

L183-187: Please rephrase these sentences by showing the list of genes and functions involved for up-regulated first and then for down-regulated genes.

L190: The abbreviation ‘DEG’ is not previously defined in the manuscript.

L191: Please clarify what this means by DEGs being considered a class. I know that it means grouping variants into two classes, DEGs and non-DEGs, but it could be more intuitive for readers.

L219: Please remove the blank space at the beginning of the paragraph, or if the authors want to use it, be consistent throughout the manuscript.

L220: Is it necessary to define lactating (L) and non-lactating (N) abbreviations if they are not used later in the manuscript?

L225-227: Please rephrase.

L257-285: Please remove these paragraphs to gain clarity. See the major comment.

L305: Please do not start the sentence with numbers or abbreviations.

L358: Please declare the abbreviation for eQTL and sQTL.

L394: Please don’t use subtitles in the discussion section.

L408: The heritability of milk yield, fat, and protein is around 0.25-0.30. Do the authors think the high heritability obtained using deregressed PTAs as phenotype impacts their results?

L466-477/ L543-546: Please remove these lines. See major comment.

L553: Please change ‘Analyzed RNA sequencing data’ to ‘RNA sequencing data.’

L574: Please change ‘Computing mammary-specific genes using t-statistic’ to ‘Mammary-specific gene expression.’

L589: Please change ‘Analysis of miRNA sequencing data’ to ‘miRNA sequencing data’ or ‘micro-RNA sequencing data’.

L591: Please change by ‘…postpartum (i.e., the peak lactation period)’.

L598: Please use the abbreviation miRNAs, or open the abbreviation to start the sentence as Micro RNAs.

L600: Please include the reference for miRanda software or pipeline.

L604-607: Please rephrase.

L605: Please be consistent with the way you write the P-value throughout the manuscript. If you use p-value, keep it the same throughout the manuscript.

L650: How did the authors deregressed the PTAs? Please specify in the text.

L668-671/ L677 / L685/ L719-721: Please use ‘;’ instead of ‘.’, such as ‘where y is the vector of deregressed PTAs; 1mu is a vector of trait means; g is the vector of total additive genetic effects ….’. Please use this format for the other equation descriptions.

L684: Why do the authors include ‘enrichment’ in bold, and in Eq. 2, ‘RNA editing level’ is not in bold format?

L699: Please do not use bolds for these subtitles, such as ‘Genomic prediction.’

L735: Please specify the software used for genomic predictions and how the models were implemented for variance component estimations.

L736-804: This part is completely disconnected from the other parts of this section. Also, it should be removed. See major comment.

References: Please check all your references, with special attention to the doi provided. Some of them don’t have the same format, and some are missing or not working.
