# Supplementary material for: Mammary gland multi-omics data reveals new genetic insights into milk production traits in dairy cattle
Source: PLoS Genet. 2025 Apr 17;21(4):e1011675. doi: 10.1371/journal.pgen.1011675 (PMC12054919; doi:10.1371/journal.pgen.1011675)
Supplement: Attachment — Submitted filename: Response to Reviewers final.docx [file pgen.1011675.s030.docx]

**Response to Reviewer 1:**

Reviewer #1: The authors present a throughout study of integrating genomics aiming to improve the GS. The collection of molecular phenotype is unique and partially been experimental validate. Even though the improvement of GS is not substantial, the knowledge and information generated during the study is also useful to the community.

Minor comments:

1. Line 30~32, the summary about selection of miRNA is not informative. It is a nice result. However, the wording did not function as abstract, since the reader have to read the result part to understand the sentence. Please rephrase it.

Response: Sorry for our unclear statement. We have rephrased this sentence. “We proposed a novel strategy for selecting candidate miRNAs by identifying overlaps that exhibit a significant number of mRNA targets with negative correlations, as well as a relatively large genetic variance explained by these targets.” at Line 30-33 Page 2.

2. Line 36, more than what?

Response: Sorry for our unclear statement. We have revised this sentence. “Furthermore, we found that mammary enhancers explained more genetic variance for milk production traits than repressive regulatory elements, while minor changes in DNA methylation accounted for more variance than larger changes.” at Line 33-35 Page 2.

3. Line 37~38, If it is routine but not the SNP panel you just mentioned, "these" is not necessary.

Response: Thanks for the reviewers’ comments. The routine SNPs were not from the new SNP panel. We have deleted “these” in this sentence at Line 37 Page 2.

4. Line 78~79, Not, this is not the argument. "Replacing the routine SNP panel with the WGS variants marginally improve the prediction accuracy."

Response: Thanks for the reviewers’ comments. We have revised this sentence. “Several strategies have been employed to replace the routine SNP panel with imputing variants, resulting in a marginal improvement in predictive accuracy [13, 14].” at Line 75-76 Page 4.

5. Line 79~81, Incomplete sentence.

Response: Thanks for the reviewers’ comments. We have revised this sentence. “One effective approach involves narrowing down the vast array of variants to a more manageable subset by focusing on functional classes known to be associated with complex traits. This approach increases the likelihood that the selected variants will significantly influence these traits.” at Line 76-79 Page 4.

6. Line 92, have not yet.

Response: Thanks for the reviewers’ comments. We have corrected it at Line 90 Page 5.

7. Line 103, facilitate.

Response: Thanks for the reviewers’ comments. We have corrected it to “These approaches facilitate the use of biological priors” at Line 95 Page 5.

8. Line 116, hypothesized.

Response: Thanks for the reviewers’ comments. We have corrected it at Line 108 Page 5.

9. Line 146~147, go to discussion.

Response: Thanks for the reviewers’ comments. We have moved the discussion sentence to the Discussion section at Line 369, Page 14.

10. Line 148~149, go to discussion.

Response: Thanks for the reviewers’ comments. We have moved the discussion sentence to the Discussion section at Line 372-373 on Page 14.

11. Line 154, estimations were

Response: Thanks for the reviewers’ comments. We have corrected it at Line 142 Page 6.

12. Line 157, More than which type of variants?

Response: Thanks for the reviewers’ comments. We have revised this sentence. “After adjusting the SNP numbers of each region, we observed that variants located in the 5'-UTR, synonymous, and splicing regions accounted for more variance than those found in intergenic regions, introns, and downstream regions” at Line 143-145 Page 6.

13. Line 220, remove the part of sentence with DESeq2, it belong to mthod.

Response: Thanks for the reviewers’ comments. We have removed it at line 208-209 Page 8.

14. Line 540, what difference and which class.

Response: Thanks for the reviewers’ comments. We have revised this sentence. “The significant variance explanation provided by various functional classes indicates that these classes contain variants capable of contributing to trait variation, highlighting the importance of prioritizing them in future studies.” at Line 474-476 Page17.

**Response to Reviewer 2:**

Reviewer #2: Attached review

I have reviewed the manuscript titled **‘Mammary gland multi-omics data reveals new genetic insights into milk production traits in dairy cattle’** by Cai et al. The manuscript is interesting. The introduction is good, easily introduces the reader to the topic, and identifies the gaps in knowledge to fill. The results and discussion sections are long and sometimes hard to follow. I have major comments for the authors.

**Major comments**

The results section is long, and sometimes, it is hard to follow the amount of results the authors showed. I propose the authors remove the section about functional verification of miRNAs that shows up in the manuscript disconnected from the other results (even if I understand the relevance of this verification), but those results contribute little to the manuscript. Also, in the materials and methods section, this functional verification is at the end and disconnected from the previous paragraph. The authors have many relevant results to emphasize, and I think that extra results only increase the complexity of the manuscript.

Response: Thanks for the reviewers’ suggestions. This version of the manuscript removes the results, discussion, and methods section about functional verification of miRNAs.

The authors used deregressed PTAs as the milk production phenotypes. I am concerned about the potential impact on the results obtained. The authors showed a large genetic variance explained for those phenotypes larger than we expected using DTD.

Response: Thanks for reviewers’ comments. The PTAs represent breeding values after removing fixed non-genetic effects, and their reliabilities were used to quantify the amount of information available for different individuals. De-regressed PTAs were calculated as described by Garrick et al., by dividing the PTA by its squared reliability, thereby excluding parental information and decreasing inter-animal dependency. It has been reported that deregressed information is used as an alternative to EBVs in genomic selection to reduce bias and increase reliability (Garrick, Taylor, & Fernando, 2009; Thistlethwaite et al., 2019). Phenotype using deregressed PTAs has been widely used for GWAS study (Fang et al., 2019; Jiang et al., 2019; Pedrosa et al., 2023) and genomic prediction study (Li et al., 2020; Paul M VanRaden & O'Connell, 2018; Paul M. VanRaden, Tooker, O’Connell, Cole, & Bickhart, 2017) in US dairy.

As for the heritability estimations of dairy traits. heritabilities of milk production traits using daughter trait deviations with 630K HD SNPs were 0.419~0.656 for 11923 Australia bulls (Table 1) (Xiang et al., 2019). In this study, our heritability (genetic variance) was 0.711~ 0.892 based on de-regressed PTAs 3.1M variants with for 23,566 US bulls. We believe that of using de-regressed PTAs is one of the main reasons to minimize biases, resulting in the high heritability estimates of milk production traits. Additionally, the increased sample size, and higher marker density of our study are also factors contributing to the calculation of high heritability.

Table 1 The reported heritability of milk production traits by Ruidong et al., 2019.

**Minor comments**

L49: Please remove ‘(eQTLs and sQTLs)’.

Response: Thanks for the reviewers’ comments. We have removed it on Line 48, Page 3.

L64: Please change ‘dairy research’ to ‘animal genetics research’ to give a broader idea of the relevance of the high-throughput technologies.

Response: Thanks for the reviewers’ comments. We have corrected it on Line 61, Page 4.

L72: Please remove the extra blank space after ‘[11],’.

Response: Thanks for the the reviewers’ comments. We have removed the extra blank space on Line 69, Page 4.

L93-99: Please remove lines 93 to 99. This part is extra information of only one of the functional classes that does not contribute much to the paragraph per se. Or, if authors want to keep it, it could be good to have more examples for different functional classes and not only for RNA editing.

Response: Thanks for the reviewers’ comments. We have removed these sentences.

L119-125: Please rewrite. I suggested the following: ‘This study of the mammary gland aims to 1) identify and define the sequencing segments …; 2) determine which functional indicators …; and 3) assess the increase in accuracy ….’.

Response: Thanks for the Reviewers’ suggestion. We have revised the objectives according to Reviewers’ suggestion. “This study of the mammary gland aims to 1) identify and define the sequencing segments and candidate biological molecules, such as coding/non-coding genes, RNA editing, DNA methylation, and histone modification, using mammary multi-omics data; 2) determine which functional indicators are most effective in predicting sequence variants with the highest probability of influencing milk production traits in dairy cattle; and 3) assess the increase in accuracy of genomic predictions that can be achieved using the prior biological information, compared with that from the non-prior information strategy in the same data set.” at Line 111-117 Page 5.

L126-128: Please remove these lines. They should be placed in the discussion or conclusion sections.

Response: Thanks for the reviewers’ comments. We have removed these lines at Line 145 Page 6.

L131: Please change the subtitle ‘Genome partitioning of genetic variation by autosomes and genomic regions’ to ‘Genome partitioning of genetic variation’.

Response: Thanks for the reviewers’ comments. We have corrected it at Line 121 Page 6.

L146-149/ L290-291: Please do not include any references in the results section. These lines should be in the discussion section.

Response: Thanks for the reviewers’ comments. We have moved the discussion sentences to Discussion section at Line 372-373 Page 14 and Line 418-419 Page 15.

L168: Please remove the extra punctuation you have in ‘(Figure. S3)’.

Response: Thanks for the reviewers’ comments. We have removed the extra dot at Line 157 Page 7.

L170-172: These line could be shortened by combine in one like this ‘As expected, the lactating mammary gland-specific genes explained more genetic variance of milk production (MFP and MPP) than the other four mammary tissues (Table 1). Otherwise, in non-mammary tissues, …’.

Response: Thanks for the reviewers’ comments. We have revised this sentence according to reviewer’s suggestion at Line 159-161 Page 7.

L183-187: Please rephrase these sentences by showing the list of genes and functions involved for up-regulated first and then for down-regulated genes.

Response: Thanks for the reviewers’ comments. We have revised these sentences according to reviever’s suggestion at Line 167-175 Page 7.

L190: The abbreviation ‘DEG’ is not previously defined in the manuscript.

Response: Thanks for the reviewers’ comments. We have added the full names of DEGs at Line 178, Page 7.

L191: Please clarify what this means by DEGs being considered a class. I know that it means grouping variants into two classes, DEGs and non-DEGs, but it could be more intuitive for readers.

Response: Thanks for the reviewers’ comments. We have revised this sentence. “To assess the contribution of genetic variance by differentially expressed genes (DEGs), we classified variants into two groups based on whether they were located within DEGs or their flanking 5 kb regions, or not. We applied a similar classification strategy for non-DEGs.” at Line 178-180 Page 7.

L219: Please remove the blank space at the beginning of the paragraph, or if the authors want to use it, be consistent throughout the manuscript.

Response: Thanks for the reviewers’ comments. We have removed the blank space at Line 208, Page 8.

L220: Is it necessary to define lactating (L) and non-lactating (N) abbreviations if they are not used later in the manuscript?

Response: Thanks for the reviewers’ comments. We have removed (L) and (N) at Line 208-209 Page 8.

L225-227: Please rephrase.

Response: Thanks for the reviewers’ comments. We have revised this sentence “Our analysis indicated that 53 miRNAs exhibited a significant number of mRNA targets with negative correlations (Fisher’s exact test P< 0.05, Table S5)” at Line 213-214 Page 8.

L257-285: Please remove these paragraphs to gain clarity. See the major comment.

Response: Thanks for the reviewers’ comments. We have these paragraphs.

L305: Please do not start the sentence with numbers or abbreviations.

Response: Thanks for the reviewers’ comments. We revised this sentence using “41.4% of RNA editing sites were detected across multiple samples” at Line 263 Page 10.

L358: Please declare the abbreviation for eQTL and sQTL.

Response: Thanks for the reviewers’ comments. We have added the abbreviations for eQTL and sQTL at Line 313, Page 12.

L394: Please don’t use subtitles in the discussion section.

Response: Thanks for the reviewers’ comments. We have removed all subtitles in the discussion section.

L408: The heritability of milk yield, fat, and protein is around 0.25-0.30. Do the authors think the high heritability obtained using deregressed PTAs as phenotype impacts their results?

Response: Thanks for the reviewers’ comments. According to previous heritability estimation of Canada (Oliveira Junior et al., 2021) and New Zealand Holstein (Jayawardana, Lopez-Villalobos, McNaughton, & Hickson, 2023) population study, the heritability of yield traits (milk, fat, protein) was around 0.3, while the heritability of fat and protein percentage was around 0.6. Both these two studies used the original phenotype based on female cows. For the US dairy population, due to privacy concerns of breeding farms or other commercial reasons, the original phenotypic data of the cows in CDCB cannot be available for research. The phenotypes used in this study are derived from bulls' PTAs (Predicted Transmitting Abilities), which are calculated based on the milk production performance of many progenies, resulting in breeding values with higher reliability. This reliability is notably higher compared to breeding values directly estimated from cow phenotypes. We can also observe similar differences in the reliability of genomic predictions. The reliability of genomic predictions for US dairy bulls typically ranges from 0.6 to 0.8 (Paul M. VanRaden et al., 2017), while the reliability of genomic predictions based on dairy cows usually ranges from 0.3 to 0.6 (Brøndum et al., 2015; Oliveira et al., 2019).

L466-477/ L543-546: Please remove these lines. See major comment.

Response: Thanks for the reviewers’ suggestions. We have removed the experimental validation of miRNAs in discussions and conclusion.

L553: Please change ‘Analyzed RNA sequencing data’ to ‘RNA sequencing data.’

Response: Thanks for the reviewers’ comments. We have revised it at Line 484 Page 17.

L574: Please change ‘Computing mammary-specific genes using t-statistic’ to ‘Mammary-specific gene expression.’

Response: Thanks for the reviewers’ comments. We have revised it at Line 505 Page 18.

L589: Please change ‘Analysis of miRNA sequencing data’ to ‘miRNA sequencing data’ or ‘micro-RNA sequencing data’.

Response: Thanks for the reviewers’ comments. We have revised it at Line 520 Page 19.

L591: Please change by ‘…postpartum (i.e., the peak lactation period)’.

Response: Thanks for the reviewers’ comments. We have revised it at Line 522 Page 19.

L598: Please use the abbreviation miRNAs, or open the abbreviation to start the sentence as Micro RNAs.

Response: Thanks for the reviewers’ comments. We have revised it at Line 529 Page 19.

L600: Please include the reference for miRanda software or pipeline.

Response: Thanks for the reviewers’ comments. We have added the reference and software version at Line 531-532 Page 19

L604-607: Please rephrase.

Response: Thanks for the reviewers’ comments. We rewrote this sentence in Lines 532-538, Page 19.

L605: Please be consistent with the way you write the P-value throughout the manuscript. If you use a p-value, keep it the same throughout the manuscript.

Response: Thanks for the reviewers’ comments. We revised them and used a uniform P-value throughout the manuscript.

L650: How did the authors deregressed the PTAs? Please specify in the text.

Response: Thanks for the reviewers’ comments. We added the procedure of deregressed PTAs in the Method section: “All the 23,566 Holstein bulls used in this study had highly reliable PTAs (predicted transmitting abilities) for 12 production and reproduction traits, which have been described in previous studies (Jiang et al., 2019; Paul M. VanRaden et al., 2017). The PTAs represent breeding values after removing fixed non-genetic effects, and their reliabilities were used to quantify the amount of information available for different individuals (P. M. VanRaden & Wiggans, 1991). De-regressed PTAs were calculated, as described by Garrick et al., dividing the PTA by its squared reliability, thereby excluding parental information and decreasing inter-animal dependency (Garrick et al., 2009). These de-regressed PTAs were then used as the phenotype in subsequent analyses.” at Line 583 -590 Page 21.

L668-671/ L677 / L685/ L719-721: Please use ‘;’ instead of ‘.’, such as ‘where y is the vector of deregressed PTAs; 1mu is a vector of trait means; g is the vector of total additive genetic effects ….’. Please use this format for the other equation descriptions.

Response: Thanks for the reviewers’ comments. We revised them according to reviewer’s suggestion at Line 604-606, 613-615 and 623-624 Page 22.

L684: Why do the authors include ‘enrichment’ in bold, and in Eq. 2, ‘RNA editing level’ is not in bold format?

Response: We're sorry for our uncorrected bold use. We revised ‘Enrichments’ with regular style at Line 622, Page 22.

L699: Please do not use bolds for these subtitles, such as ‘Genomic prediction.’

Response: Thanks for the reviewers’ comments. We revised it at Line 637 Page 23..

L735: Please specify the software used for genomic predictions and how the models were implemented for variance component estimations.

Response: Thanks for the reviewers’ comments. Both GBLUP and MultiBLUP models were implemented using LDAK v5.2 software. The BayesR and BayesRC models were implemented using BayesRv2 and BayesRCO software, respectively. Both software programs employed the same parameters, with a total of 25,000 MCMC iterations, of which the first 5,000 iterations were discarded as burn-in. The GRM for each class was calculated, and a REML analysis was performed using GCTA by simultaneously fitting the GRM for all classes in the model to estimate the phenotypic variance for each class. The variance proportion ($\boldsymbol{h}_{\boldsymbol{f}}^{\mathbf{2}}$) for each class was calculated as the ratio of the class variance to the total variance. We added the above information in the manuscript at Lines 661-662 and 674-677.

L736-804: This part is completely disconnected from the other parts of this section. Also, it should be removed. See major comment.

Response: Thanks for the reviewers’ suggestions. We have removed them.

References: Please check all your references, with special attention to the doi provided. Some of them don’t have the same format, and some are missing or not working.

Response: Thanks for the reviewers’ suggestions. We have checked all references and corrected them.

**Response to Reviewer 3:**

Reviewer #3: This is a very interesting and well written manuscript. I agree with the author's claim in the title that new insights into the genetics of milk production are generated. However, I do not find the improvement of reliabilities very impressive. I only have one concern related to the description of the methodlogy, especially the data sources. The design is complex using various data. The reader might get lost. I think a graphical overview on the workflow and data would be appreciated. This will also improve the understanding of the results. When e.g. it is stated that there is a efect of particular miRNAs on protein content it would mean that there is a relationship between these miRNA and protein content PTAs, right?

Response: Thank you for the reviewers’ valuable suggestions. Although the genomic selection accuracy in our study did not show a significant improvement, this is primarily because the genotype and phenotype datasets used in dairy cattle breeding have already undergone extensive optimization, resulting in a high baseline level of prediction reliability. Achieving further substantial improvements under such conditions is inherently challenging. Nevertheless, our study is based on extensive milk production data from USDA bulls rather than foundational cow data. Even a modest 0.1% improvement in genomic prediction accuracy holds considerable significance for the dairy industry.

As recommended by the reviewer, we have incorporated a graphical overview in Figure 1 (see below) to enhance the clarity and comprehension of our results.

Deregressed PTAs were used as phenotypes in the variance component analysis, as they exclude parent information and reduce dependency among animals (Garrick et al., 2009). If the targets of a particular miRNA explain a significant portion of the variance in protein yield, it suggests that this miRNA may play a role in regulating protein content.

Figure 1. Overview of the analysis. The functional classes, including mammary-specific genes, lactation-associated genes, novel long non-coding RNAs, miRNAs, RNA editing sites, DNA methylation, histone modifications, expression quantitative trait loci (QTL), and splicing QTL, were defined using mammary multi-omics data. Variance component analysis of milk production traits was performed based on variants split using GREML in 23,566 Holstein bulls. Two strategies for genotype modification were applied to improve the reliability of genomic predictions.

Furthermore, the introduction, although very nicely written, could be shortened a bit.

Response: Thanks for the reviewers’ suggestions.

We have streamlined the introduction section by removing less relevant content, such as the definition and function of RNA editing. Additionally, we have revised the final paragraph outlining the research objectives to make it more concise and clearer.

Have all data underlying the figures and results presented in the manuscript been provided?

Large-scale datasets should be made available via a public repository as described in the PLOS Genetics data availability policy, and numerical data that underlies graphs or summary statistics should be provided in spreadsheet form as supporting information.

Response: Thanks for the reviewers’ remind. We uploaded all data for figures and mammary multi-omics results on FigShare (<https://doi.org/10.6084/m9.figshare.27991175.v>1).

Finally, we wish to thank editor and reviewers again for the critical comments and valuable suggestions. If you have any question on this manuscript, please feel free to contact me. Thank you again for your time and favorable consideration.

Sincerely,

Jiuzhou Song, [Songj88@umd.edu](mailto:Songj88@umd.edu)

University of Maryland

**Reference:**

Brøndum, R. F., Su, G., Janss, L., Sahana, G., Guldbrandtsen, B., Boichard, D., & Lund, M. S. (2015). Quantitative trait loci markers derived from whole genome sequence data increases the reliability of genomic prediction. *Journal of Dairy Science, 98*(6), 4107-4116. doi:<https://doi.org/10.3168/jds.2014-9005>

Fang, L., Jiang, J., Li, B., Zhou, Y., Freebern, E., Vanraden, P. M., . . . Ma, L. (2019). Genetic and epigenetic architecture of paternal origin contribute to gestation length in cattle. *Communications Biology, 2*(1), 100. doi:10.1038/s42003-019-0341-6

Garrick, D. J., Taylor, J. F., & Fernando, R. L. (2009). Deregressing estimated breeding values and weighting information for genomic regression analyses. *Genetics Selection Evolution, 41*(1), 55. doi:10.1186/1297-9686-41-55

Jayawardana, J. M. D. R., Lopez-Villalobos, N., McNaughton, L. R., & Hickson, R. E. (2023). Heritabilities and genetic and phenotypic correlations for milk production and fertility traits of spring-calved once-daily or twice-daily milking cows in New Zealand. *Journal of Dairy Science, 106*(3), 1910-1924. doi:<https://doi.org/10.3168/jds.2022-22431>

Jiang, J., Cole, J. B., Freebern, E., Da, Y., VanRaden, P. M., & Ma, L. (2019). Functional annotation and Bayesian fine-mapping reveals candidate genes for important agronomic traits in Holstein bulls. *Communications Biology, 2*(1), 212. doi:10.1038/s42003-019-0454-y

Li, B., VanRaden, P. M., Guduk, E., O'Connell, J. R., Null, D. J., Connor, E. E., . . . Cole, J. B. (2020). Genomic prediction of residual feed intake in US Holstein dairy cattle. *Journal of Dairy Science, 103*(3), 2477-2486. doi:<https://doi.org/10.3168/jds.2019-17332>

Oliveira, H. R., Lourenco, D. A. L., Masuda, Y., Misztal, I., Tsuruta, S., Jamrozik, J., . . . Schenkel, F. S. (2019). Application of single-step genomic evaluation using multiple-trait random regression test-day models in dairy cattle. *Journal of Dairy Science, 102*(3), 2365-2377. doi:<https://doi.org/10.3168/jds.2018-15466>

Oliveira Junior, G. A., Schenkel, F. S., Alcantara, L., Houlahan, K., Lynch, C., & Baes, C. F. (2021). Estimated genetic parameters for all genetically evaluated traits in Canadian Holsteins. *Journal of Dairy Science, 104*(8), 9002-9015. doi:10.3168/jds.2021-20227

Pedrosa, V. B., Boerman, J. P., Gloria, L. S., Chen, S.-Y., Montes, M. E., Doucette, J. S., & Brito, L. F. (2023). Genomic-based genetic parameters for milkability traits derived from automatic milking systems in North American Holstein cattle. *Journal of Dairy Science, 106*(4), 2613-2629. doi:<https://doi.org/10.3168/jds.2022-22515>

Thistlethwaite, F. R., Ratcliffe, B., Klápště, J., Porth, I., Chen, C., Stoehr, M. U., & El-Kassaby, Y. A. (2019). Genomic selection of juvenile height across a single-generational gap in Douglas-fir. *Heredity, 122*(6), 848-863. doi:10.1038/s41437-018-0172-0

VanRaden, P. M., & O'Connell, J. R. (2018). Validating genomic reliabilities and gains from phenotypic updates. *Interbull Bulletin*(53).

VanRaden, P. M., Tooker, M. E., O’Connell, J. R., Cole, J. B., & Bickhart, D. M. (2017). Selecting sequence variants to improve genomic predictions for dairy cattle. *Genetics Selection Evolution, 49*(1), 32. doi:10.1186/s12711-017-0307-4

VanRaden, P. M., & Wiggans, G. R. (1991). Derivation, calculation, and use of national animal model information. *J Dairy Sci, 74*(8), 2737-2746. doi:10.3168/jds.S0022-0302(91)78453-1

Xiang, R., Berg, I. v. d., MacLeod, I. M., Hayes, B. J., Prowse-Wilkins, C. P., Wang, M., . . . Goddard, M. E. (2019). Quantifying the contribution of sequence variants with regulatory and evolutionary significance to 34 bovine complex traits. *Proceedings of the National Academy of Sciences, 116*(39), 19398-19408. doi:doi:10.1073/pnas.1904159116
