# Supplementary material for: Mammary gland multi-omics data reveals new genetic insights into milk production traits in dairy cattle
Source: PLoS Genet. 2025 Apr 17;21(4):e1011675. doi: 10.1371/journal.pgen.1011675 (PMC12054919; doi:10.1371/journal.pgen.1011675)
Supplement: Attachment — Submitted filename: Response to Reviewers-Jan-3.docx [file pgen.1011675.s031.docx]

**Editor Comments**:

Before we can send back the manuscript to the original reviewers, we would like the authors to address our concerns about data and code sharing:

1. In the data availability section, explicitly state where the genotype data can be downloaded from, also where the phenotype (milk yield) can be obtained from.

Response: Thanks for the Editor’s comments. We acknowledge the importance of providing explicit information regarding the availability of genotype and phenotype data. However, the genotype and phenotype data are owned by third parties and managed by the Council on Dairy Cattle Breeding (CDCB). To obtain access for research purposes, requests can be sent to João Dürr, CDCB Chief Executive Officer ([joao.durr@cdcb.us](mailto:joao.durr@cdcb.us)). Alternatively, you can contact the CDCB at 301-712-9339 or visit their website at https://uscdcb.com/contact/ for data use. We will revise the Data Access section of the manuscript to clearly state these points and provide guidance on requesting access to the data.

2. While there is a code provided for the permutation procedure (https://github.com/WentaoCai/Permutation) described in Eq (6), the whole analysis flow is not described in code pipeline. Please provide code to reproduce the whole pipeline, not only a small part of it. Of course, the code can contain system commands to launch GCTA with explicit flags, methylation data processing, RNA editing, DESeq2 pipeline, RNAseq data processing, genotype data processing (PLINK commands), LDAK commands, etc.

Response: Thanks for the Editor’s comments. The entire pipeline, which includes RNA-seq, DESeq2, lncRNA identification, small RNA-seq, RNA editing, DNA methylation, ChIP-seq, variance component analysis, and genomic prediction, has been uploaded to our GitHub repository ([https://github.com/WentaoCai/Multi_Omics_Insights_into_Milk_Production_traits/](https://github.com/WentaoCai/Multi_Omics_Insights_into_Milk_Production_traits/)). We have also mentioned this link in the Data Access section of the manuscript.

**Figure resubmission:**

While revising your submission, please upload your figure files to the Preflight Analysis and Conversion Engine (PACE) digital diagnostic tool, https://pacev2.apexcovantage.com/. PACE helps ensure that figures meet PLOS requirements. To use PACE, you must first register as a user. Registration is free. Then, login and navigate to the UPLOAD tab, where you will find detailed instructions on how to use the tool. If you encounter any issues or have any questions when using PACE, please email PLOS at figures@plos.org. Please note that Supporting Information files do not need this step. If there are other versions of figure files still present in your submission file inventory at resubmission, please replace them with the PACE-processed versions.

Response: Thanks for the Editor’s comments. All figures were processed by PACE and resubmitted in this version of the manuscript. Please check that.

**Reproducibility:**

To enhance the reproducibility of your results, we recommend that authors of applicable studies deposit laboratory protocols in protocols.io, where a protocol can be assigned its own identifier (DOI) such that it can be cited independently in the future. Additionally, PLOS ONE offers an option to publish peer-reviewed clinical study protocols. Read more information on sharing protocols at <https://plos.org/protocols?utm_medium=editorial-email&utm_source=authorletters&utm_campaign=protocols>

Response: Thank you for your insightful suggestion regarding the deposition of laboratory protocols in protocols.io. We appreciate the emphasis on enhancing the reproducibility of research findings. However, we would like to clarify that our submitted manuscript primarily focuses on data analysis rather than experimental procedures, with minimal discussion of experimental protocols. Additionally, all relevant data analysis methods and workflows have already been made publicly available in our GitHub repository (<https://github.com/WentaoCai/Multi_Omics_Insights_into_Milk_Production_traits/>). Nevertheless, we will consider your recommendation for future submissions where applicable.
