# Supplementary material for: Mammary gland multi-omics data reveals new genetic insights into milk production traits in dairy cattle
Source: PLoS Genet. 2025 Apr 17;21(4):e1011675. doi: 10.1371/journal.pgen.1011675 (PMC12054919; doi:10.1371/journal.pgen.1011675)
Supplement: Attachment — Submitted filename: Response.docx [file pgen.1011675.s032.docx]

PGENETICS-D-24-00953R2
Mammary Gland Multi-Omics Data Reveals New Genetic Insights into Milk Production Traits in Dairy Cattle

**Reviewers' comments:**

Reviewer's Responses to Questions

**Comments to the Authors:
Please note here if the review is uploaded as an attachment.**

Reviewer #1: The authors has addressed all the concern from me.
I have one more additional comment about the abstract.

After the revision, the abstract still difficult to follow. The sentence is swiching among three marjor categories of their results: functional intepretation, method/strategy develped and the contribution to the GS. I would like to ask the authors to reorganize the sentence to describe one at a time and the flow could follow above mentioned order.

Response: Thank you for reviewers’ valuable suggestion regarding the abstract. We appreciate your suggestion to improve clarity and organization. To address your concerns, we will reorganize the abstract to clearly delineate each of the three major categories: functional interpretation, the methods and strategies developed, and the contributions to genetic selection (GS). By structuring these sections sequentially, we will enhance the flow and readability, allowing readers to better grasp our findings and their implications. Please check the Abstract section at Line 19-46 Page 2.

Reviewer #2: I have reviewed the manuscript titled ‘Mammary gland multi-omics data reveals new genetic insights into milk production traits in dairy cattle’ by Cai et al. I would like to thank the authors for addressing all my comments.
The model definition for the variance component analysis needs to be improved; it is hard to follow the different models the authors performed in the text and tables (Tables 1 and 2).

Response: We sincerely appreciate the reviewer's insightful feedback regarding the clarity of model definitions in variance component analysis and Tables 1-2. The variance in Table 1 was calculated using two genomic relationship matrices (GRMs) models, which simultaneously fitted a GRM for the functional class and a GRM for the non-functional class. However, the variance in Table 2 using ten genomic relationship matrices (GRMs) model, which simultaneously fits all nine functional classes (DEGs, lncRNAs, miRNAs, RNA editing, DNA methylation, histone modifications, eQTLs, sQTLs, splicing variants) along with one non-functional class and their respective GRMs. We have rephrased the title of Tables 1 and 2, Page 34. We described the definitions of two GRM models and ten GRM models in the method section on Lines 621-626, Page 22.

Minor comments
L74: Please remove the double comma.

Response: Thanks for the reviewers’ suggestion. We have corrected it on Line 82, Page 4.

L93-94: Please add blank spaces before the reference (“BayesRC [26], MultiBLUP [27]”).

Response: Thanks for the reviewers’ suggestion. We have corrected it on Line 101, Page 5.

L435: Please rephrase, starting the sentence with “Histone modifications such as …”

Response: Thanks for the reviewers’ suggestion. We have rephrased this sentence at Line 442, Page 16.
L520: Please add a comma after “i.e.,” and it should be in italics (idem for throughout the text).

Response: Thanks for the reviewers’ suggestion. We have corrected it on Line 528, Page 19.

L604: Please use the same notation: error variance should be σ_ε^2

Response: Thanks for the reviewers’ suggestion. We have corrected it on Line 612, Page 21.

L601-613: It is unclear why the authors used model 3 and the difference with model 4. I understand that the authors computed different GRMs and, using model 4, calculated the variance component for each functional class. Was the model 3 was used as a reference? Please clarify in the text.

Response: Thank you for your valuable feedback regarding models 3 and 4. We appreciate your attention to this detail. To clarify, model 3 was utilized to assess the overall variance explained by the genomic relationship matrices (GRMs) constructed for all genotype variants. We described these results in the Results section on lines 131-134 of page 6. Model 4 was then applied to compute each functional class's variance components using their specific GRMs. Model 3 serves as a foundational model to help readers understand the context of Model 4. To reduce potential misunderstandings, we have revised the relevant sentences on lines 609-610 of page 21 and 620-626 and 631-632 of page 22.

L605-606: Please rephrase as follows: “The heritability (h^2=(σ_g^2)/(σ_p^2 )) was the proportion of phenotypic variance (σ_p^2) explained by all variants together.”

Response: Thanks for the reviewers’ suggestion. We have corrected it on Lines 613-614, Page 22.

L614-616: This explanation should be before model 3 and clarify the value of using this model (h_all^2 comes from model 3?).

Response: Thanks for the reviewers’ suggestion. We moved and rephrased this sentence before model 3 at Line 606-608, Page 21. Sorry for our unclear statement at Line 631. Here, the $h_{all}^{2}$ has not come from model 3. It come from model 4. $h_{all}^{2}$ denotes the proportion of phenotypic variance explained by the total sum of variance from all functional classes combined from model 4. We have rephrased this sentence at Line 631 Page 22.

L621: Please check if the abbreviation should be in bold. Usually, only vectors and matrices are in bold.

Response: Thank you for the reviewers' suggestion. We have carefully checked all the letter symbols in the formulas to ensure that only vectors and matrices are in bold.

L661: Please rephrase as follows: “We performed BayesR and BayesRC using the following model:”

Response: Thanks for reviewers’ suggestion. We have corrected it at Line 671 Page 24.

L664: Please add a blank space after the comma.

Response: Thanks for reviewers’ suggestion. We have corrected it at Line 674 Page 24.

L677-687: This paragraph should be removed because it is not considered anymore in the results and discussion.

Response: Thanks for reviewers’ suggestion. We have removed it at Line 677 Page 24.

Table 2: Please check the total values. Some of them are not the exact sum of each row.

Response: Thank you for the reviewers' comments. We have carefully reviewed Table 2. Due to the overlap of some variants among functional classes, the sum of SNP numbers across all functional classes exceeds the total number of genotype variants. To clarify this, we have added a note at the bottom of Table 2. The discrepancy between the summed variances for each functional class of MY (Total: 0.738 vs. sum: 0.737) and MPY (Total: 0.736 vs. sum: 0.737) and their total item data arises from rounding within each functional class, which is considered a normal occurrence.

**Have all data underlying the figures and results presented in the manuscript been provided?**
Large-scale datasets should be made available via a public repository as described in the *PLOS Genetics* [data availability policy](http://journals.plos.org/plosgenetics/s/data-availability), and numerical data that underlies graphs or summary statistics should be provided in spreadsheet form as supporting information.

Reviewer #1: **No:** The genotype and phenotype data is still not public avaliable.

Response: Thanks for reviewers’ comments. We acknowledge the importance of providing explicit information regarding the availability of genotype and phenotype data. However, the genotype and phenotype data are owned by third parties and managed by the Council on Dairy Cattle Breeding (CDCB). To obtain access for research purposes, requests can be sent to João Dürr, CDCB Chief Executive Officer (joao.durr@cdcb.us). Alternatively, you can contact the CDCB at 301-712-9339 or visit their website at https://uscdcb.com/contact/ for data use. We will revise the Data Access section of the manuscript to clearly state these points and provide guidance on requesting access to the data.

Reviewer #2: Yes
