# Supplementary material for: Mammary gland multi-omics data reveals new genetic insights into milk production traits in dairy cattle
Source: PLoS Genet. 2025 Apr 17;21(4):e1011675. doi: 10.1371/journal.pgen.1011675 (PMC12054919; doi:10.1371/journal.pgen.1011675)
Supplement: Attachment — Submitted filename: Response_auresp_4.docx [file pgen.1011675.s033.docx]

**Additional Editor Comments:**

\## Main comments

- The Abstract still requires careful editing, in particular:

line 20-21: *defined functional classes* , what do you mean by "define", functional classes of what objects / elements ? please be more specific
Response: Thanks for the editor’s comments. “defined” was changed to “identified” at Line 20.

line 26: *Mammary specific genes* : specify what is meant by this
Response: Thanks for the editor’s comments. “Mammary specific genes” was changed to “Genes specifically expressed in the mammary gland” at Line 26.

line 28: *Differential expression* between what ?
Response: Thanks for the editor’s comments. “Differential expression analysis identified 8,560 lactation-associated genes” was changed to “We identified 8,560 differentially expressed genes (DEGs) between lactating and non-lactating tissue” at Line 29-30.

line 30: *exhibiting enriched genetic variance* not clear what this means
Response: Thanks for the editor’s comments. We have revised this sentence at Line 30-31. “with up-regulated and small-fold changes of down-regulated DEGs exhibiting enriched genetic variance.” => “Among these, both up-regulated and small-fold changes of down-regulated DEGs exhibited greater genetic variance enrichment of milk production traits than other genes”

line 32 *DNA methylation changes <0.2 contribute the most variance* please rephrase

Response: Thanks for the editor’s comments. We have revised this sentence at Line 33-34. “DNA methylation changes <0.2 contribute the most variance” => “small changes in DNA methylation level (≤0.2) contribute more variance than larger changes (> 0.2)” .

lines 34: ~~compared to randomly shifted sites~~ replace by *than expected by chance*

Response: Thanks for the editor’s comments. We have revised this sentence at Line 36.

line 35: *candidate miRNAs* candidate for what ?
Response: Thanks for the editor’s comments. “We have revised this sentence at Line 37. “selecting candidate miRNAs” => “selecting candidate miRNAs related to milk production traits”.

lines 35-37: *identifying overlaps ... explained by these targets* in the context of the abstract this sentence cannot be understood, please simplify
Response: Thanks for the editor’s comments. We rephased this sentence at Line 37-39. “by identifying overlaps that exhibit a considerable number of mRNA targets with negative correlations, along with a comparatively large genetic variance explained by these targets” => “based on the overlaps between significant enrichment tests of miRNA target correlations and the relatively large genetic variance explained by these targets.”

line 37: *a functional annotation framework* what do you mean ?
Response: Thanks for the editor’s comments. We have revised this sentence at Line 39-40. “Additionally, we established a functional annotation framework integrating nine biological classes (DEGs, lncRNAs, miRNAs, RNA editing, methylation, etc.)” => “Additionally, we integrated these nine functional classes into the variance component analysis simultaneously”

- A recurring problem throughout the manuscript is the confusion betwen "genetic variance", "variance" and "proportion of variance explained". These terms are not exchangeable (*e.g.* 0.2 genetic variance is not the same as a 20% proportion of genetic variance). Phenotypic variance and genetic variance are not equal etc. Please be specific in the text, figure legends, axis labels etc. as to which is the correct term.
Response: Thank you for the suggestions from the editor. Sorry for the negligence, we completely agree with the editor's perspective. The values used in this manuscript to measure the proportion of explained variance are all based on the ratio of genetic variance to phenotypic variance. When mentioning specific values, it is more appropriate to refer to the “proportion of variance” for trait. However, when we compare the genetic variance explained by different functional groups without involving specific values, genetic variance can substitute for “genetic variance”, as they pertain to the same phenotype (with the same denominator).

We carefully revised the inconsistencies related to the term "variance" in the article, retaining only two forms of expression: "proportion of variance" (when mentioning specific values) and "genetic variance" (when not mentioning specific values, just comparing between functional groups).

- In figures, when plotting the enrichment of categories, please present the result on a log-scale as an "enrichment" of 0.5 is of the same magnitude as an enrichment of 2 (see for example figure 2F, 3E, 4B etc.)

Response: Thank you for the editor's comments. We acknowledge that using a log-scale enrichment value may better represent depleted enrichment values (< 1). However, some functional classes exhibit variance in traits close to 0, resulting in their enrichment values also being close to 0. Therefore, we cannot use a log scale for these zero values (negative infinity). We would appreciate it if you could have any better suggestions to present the images, we will do best to optimize the expression.

- Please upload the computer codes in an archived form and provide the identifier from a repository such as SoftwareHeritage, Dryad or Zenodo.

Response: Thanks for the editor’s comments. The analysis codes used to generate the figures and the mammary-associated functional classes have been uploaded and published on FigShare (<https://doi.org/10.6084/m9.figshare.27991175.v2>)

- The Methods section *Mammary-specific gene expression* is not clear enough. Please recall what are the tissue samples categories and the exact null hypotheses tested.

Response: Thanks for the editor’s comments. We classified 152 tissues or cell types into 13 tissue categories based on established biological knowledge (Harhay et al. 2010). For each gene of lactating mammary tissue, the null hypothesis is that there is no significant difference in the gene expression level between lactating mammary tissue and non-mammary tissues. To make this section clearer, we restated this section at Line 519-531.

\## Specific comments

line 88-89 : "highest proportion of the variance per SNP" this is not clear, please explain
Response: Thanks for the editor’s comments. We have corrected this sentence at Line 89-90. “In case of dairy traits, variants in splice sites explained the highest proportions of the phenotypic variance for milk production traits per variant.”

line 91: *variant in functional class* rephrase
Response: Thanks for the editor’s comments. We have corrected this sentence at Line 92-94. "Based on the predicted heritability of each variant across functional and evolutionary categories, such as genomic location and selection signatures, Functional-And-Evolutionary Trait Heritability (FAETH) scores were proposed to provide effective biological priors for GWAS and genomic prediction."

line 96 : delete not

Response: Thanks for the editor’s comments. We have corrected it at Line 98.

line 98 99: rephrase

Response: Thanks for the editor’s comments. We have corrected this sentence at Line 99-100. “Integrating prior biological information into genomic prediction models, prioritizing likely causal markers, and improving prediction accuracy have been suggested.” => “It has been suggested to prioritize likely causal markers as prior information in genomic prediction models to enhance prediction accuracy”

line 109 : *be irrelevant* come from irrelevant ?

Response: Sorry for our unclear statement. “be irrelevant” should be changed to “come from irrelevant”. We have corrected this sentence at Line 109-110. “However, the magnitude of improvements to prediction accuracy was not as large as expected, mainly because the biological information may come from irrelevant tissues.”

lines 111: *tissue* delete, a tissue is not an organ

Response: Thanks for the editor’s comments. We have deleted it Line 112

line 140: *variance explained by MFP* -> variance **of** MFP (see main comment above)

Response: Thanks for the editor’s comments. We have revised this sentence at Line 140-141. “variance explained by MFP” => “particularly with 23.9% of the variance attributed to MFP”

line 145: *intergenic and intron* -> intergenic regions and introns / intergenic and intronic regions

Response: Thanks for the editor’s comments. We have changed it to “intergenic and intronic regions” at Line 146

line 154: t-statistic -> the type of statistic matters less than the null hypothesis tested. Please explain what hypotheses are tested here (see above).

Response: Thanks for the editor’s comments. For each gene in lactating mammary tissue, the null hypothesis is that there is no significant difference in the gene expression level between lactating mammary tissue and non-mammary tissues. We descripted this hypothesis in Method sections at Line 524-526.

line 159 : *We observed highly positive correlations* correlation of what ? (also line 161)
Response: Thanks for the editor’s comments. We have corrected this sentence at Line 160. “We observed highly positive correlations in the t-statistics of genes among non-lactating mammary gland, mammary parenchyma, and mammary fat pad.”

line 161-162: *We integrated tissue specific ...* this sentence is not clear, be more specific

Response: Sorry for our unclear statement. The sentence has been corrected in detail at Line 163-165. “We integrated tissue specific genes (top 10%) with milk production traits using genetic variation partitioning (Figure 3A).” => “We defined tissue-specific genes for each tested tissue based on the rank of t-statistics (top 10%). Additionally, we partitioned the variance in milk production explained by SNPs into tissue-specific genes or their flanking 5 kb regions (Figure 3A).”

line 171-175: please specificy the FDR threshold and the method used to estimate it.

Response: Thanks for the editor’s comments. We added FDR threshold and the method in this sentence at Line 175. “We identified 8,560 significantly dysregulated genes between the non-lactating period and lactation using DESeq2 with adjusted P-value < 0.05, including 3,790 up-regulated genes and 4,770 down-regulated genes in lactation”.

line 185: *explain large variance* see main comment

Response: Thanks for the editor’s comments. We have corrected this sentence at Line 188. “DEGs could explain large variance” => “DEGs could explain large proportion of variance for milk production traits”

line 190: *the other two* -> *two other*
Response: Thanks for the editor’s comments. We have corrected it at Line 194.

line 191: *according* -> for line 208-209: *The variances ...* this is not a valid sentence, please explain.

Response: Thank you for the editor's comments. The two additional groups (non-DEGs and others) described here are provided as a comparative background for the eight DEG groups. The non-DEG and other groups are also displayed in Figure 3E. This sentence will help the reader better understand the variance contribution of DEGs. To enhance the understanding of this sentence, we have revised it at Line 194-195.

“In addition, we built the other two groups according to variants located in non-DEGs or outside of genes.” => “In addition, we built two other groups based on variants located in genes that were not differential expressed (non-DEGs) or located outside of any genes (others).”

lines 215: *inverse correlation* do you mean negative?

Response: Surely, “inverse correlation” was change to “negative correlation” at Line 220.

lines 244-247: *We proposed the candidate ...* These two sentences are not clear, please rephrase.

Response: Thank you for the editor's comments. We have rephrased these sentences at Line 249-251. “We proposed the candidate miRNAs for a trait through the overlapped results between significant enrichment of targets with correlation and the relatively large genetic variance explained by their targets (h_t_^2^ > 0.1). The targets-correlation significant miRNAs were also enriched in miRNAs with relatively large genetic variance (Table S6).” => “We identified candidate miRNAs for a trait based on the overlaps between significant enrichment tests of miRNA target correlations and the relatively large proportion of variance explained by these targets. (h_t_^2^ > 0.1, Table S6).”

ine 293: *more genetic variance* than what?

Response: Thanks for the editor’s comments. We haved corrected this sentence at Line XXX. “RNA editing captured more genetic variance for MFP and MPP” => “RNA editing captured more genetic variance for MFP and MPP compared to other milk production traits.”

line 384 *more closed* closer?

Response: Thanks for the editor’s comments. We have corrected it at Line 407.

line 387: *milk cell is moderately correlated with lactating mammary gland based on their t-statistics* I don't understand this sentence, please explain and rephrase.
Response: Sorry for our unclear statement. We have rephrased this sentence at Line 410-412. “Milk cell is moderately correlated with lactating mammary gland based on their gene specificity value (t-statistics), implying that somatic milk cells can serve as an indicator for studying gene expression in lactating mammary tissue but cannot fully replace it.”

line 391: *The liver captured considerable variance* The organ certainly does not. What do you mean?

Response: Sorry for our unclear statement. We have rephrased this sentence at Line 413-415. “Moreover, the specifically expressed genes in the lactating mammary gland captures more genetic variance of milk production traits than those in the other four mammary tissues. The specifically expressed genes in the liver captured considerable variance for milk production traits, indicating its active and complex functions in synthesizing proteins and fat.”

line 397: *immune, disease resistant* -> *immunity, disease resistance*

Response: Thanks for the editor’s comments. We haved corrected it at Line 421-422.

line 403-404: *thousands of lncRNAs are dynamic changes* please rephrase, this does not make sense

Response: Thanks for the editor’s comments. We have corrected this sentence at Line 428. “thousands of lncRNAs are dynamic changes during lactation” => “thousands of lncRNAs are differentially expressed during lactation”

line 408: *As the simple target ...* This sentence is not finished
Response: Thanks for the editor’s comments. We have corrected this sentence at Line 433-434. “As the simple target prediction algorithms of miRNA may generate a large portion of false positive miRNA targets” => “As the simple target prediction algorithms of miRNA may generate a large portion of false positive miRNA targets, it is important to use complementary approaches that integrate expression data to enhance the accuracy of predicted miRNA-mRNA interactions.”

line 525: *MicoRNA* -> MicroRNA

Response: Thanks for the editor’s comments. We have corrected it at Line 558.
